# Core–periphery structure in directed networks

**DOI:** 10.1098/rspa.2019.0783

**Published:** 2020-09-09

**Authors:** Andrew Elliott, Angus Chiu, Marya Bazzi, Gesine Reinert, Mihai Cucuringu

**Affiliations:** 1 The Alan Turing Institute, London, UK; 2 Department of Statistics, University of Oxford, Oxford, UK; 3 Mathematical Institute, University of Oxford, Oxford, UK; 4 Mathematics Institute, University of Warwick, Coventry, UK

**Keywords:** core–periphery, spectral methods, low-rank approximation, directed networks

## Abstract

Empirical networks often exhibit different meso-scale structures, such as community and core–periphery structures. Core–periphery structure typically consists of a well-connected core and a periphery that is well connected to the core but sparsely connected internally. Most core–periphery studies focus on undirected networks. We propose a generalization of core–periphery structure to directed networks. Our approach yields a family of core–periphery block model formulations in which, contrary to many existing approaches, core and periphery sets are edge-direction dependent. We focus on a particular structure consisting of two core sets and two periphery sets, which we motivate empirically. We propose two measures to assess the statistical significance and quality of our novel structure in empirical data, where one often has no ground truth. To detect core–periphery structure in directed networks, we propose three methods adapted from two approaches in the literature, each with a different trade-off between computational complexity and accuracy. We assess the methods on benchmark networks where our methods match or outperform standard methods from the literature, with a likelihood approach achieving the highest accuracy. Applying our methods to three empirical networks—faculty hiring, a world trade dataset and political blogs—illustrates that our proposed structure provides novel insights in empirical networks.

## 1. Introduction

Networks provide useful representations of complex systems across many applications [1], such as physical, technological, information, biological, financial and social systems. A network in its simplest form is a graph in which nodes represent entities and edges represent pairwise interactions between these entities. In this paper, we consider directed unweighted networks.

Given a network representation of a system, it can be useful to investigate the so-called meso-scale features that lie between the micro-scale (local node properties) and the macro-scale (global network properties). Typical meso-scale structures are community structure (by far the most commonly studied), core–periphery structure, role structure and hierarchical structure [1–3]; often, more than one of these is present in a network (see for example [2] or [4]).

Here we focus on core–periphery structure. The concept of core–periphery structure was first formalized by Borgatti & Everett [5]. Typically, core–periphery structure is a partition of an undirected network into two sets, a *core* and a *periphery*, such that there are dense connections within the core and sparse connections within the periphery. Furthermore, core nodes are reasonably well connected to the periphery nodes [5]. Extensions allow for multiple core–periphery pairs and nested core–periphery structures [2,4,6]. Algorithms for detecting (different variants) of core–periphery structure include approaches based on the optimization of a quality function [2,5,7–9], spectral methods [10–12] and notions of core–periphery based on transport (e.g. core nodes are likely to be on many shortest paths between other nodes in the network) [12,13]. Core–periphery detection has been applied to various fields such as economics, sociology, international relations, journal-to-journal networks and networks of interactions between scientists; see [14] for a survey.

Many methods for detecting core–periphery structure were developed for undirected networks. Although these can be (and in some cases have been) generalized to directed graphs, they do not also generalize the definition of a discrete core and periphery to be edge-direction dependent, but, rather, either disregard the edge direction or consider the edge in each direction as an independent observation [2,5,15,16], or use a continuous structure [17]. A notable exception is [18], but with a different notion of core than the one pursued here. The discrete structure which is most closely related to our notion of directed core–periphery structure is the bow-tie structure [19,20]. Bow-tie structure consists of a core (defined as the largest strongly connected component), an in-periphery (all nodes with a directed path to a node in the core), an out-periphery (all nodes with a directed path from a node in the core) and other sets containing any remaining nodes [20–22].

In this paper, we propose a generalization of the block model introduced in [5] to directed networks, in which the definition of both core and periphery are edge-direction dependent. Moreover, we suggest a framework for defining cores and peripheries in a way that accounts for edge direction, which yields as special cases a bow-tie-like structure and the structure we focus on in the present paper. Our accompanying technical report explores a small number of additional methods [23]. Extensions to continuous formulations (e.g. as in [24]) or multiple types of meso-scale structure are left to future work.

We suggest three methods to detect the proposed directed core–periphery structure, which each have a different trade-off between accuracy and computational complexity. The first two methods are based on the Hyperlink-Induced Topic Search (HITS) algorithm [25] and the third on likelihood maximization. We illustrate the performance of methods on synthetic and empirical networks. Our comparisons with bow-tie structure illustrate that the structure we propose yields additional insights about empirical networks. Our main contributions are (i) a novel framework for defining cores and peripheries in directed networks; (ii) scalable methods for detecting these structures; (iii) a comparison of said methods; and (iv) a systematic approach to method selection for empirical data.

This paper is organized as follows. In §2, we consider directed extensions to the classic core–periphery structure. We introduce a novel block model for directed core–periphery structure that consists of four sets (two periphery sets and two core sets) and a two-parameter synthetic model that can generate the proposed structure. In electronic supplementary material, A, we consider alternative formulations. We further introduce a pair of measures to assess the quality of a detected structure; the first one is a test of statistical significance, and the second one is a quality function that enables comparison between different (statistically significant) partitions. In §3, we introduce three methods for detecting the proposed directed core–periphery structure. Section 4 illustrates the performance of our methods on synthetic benchmark networks, and validates the use of our proposed partition quality measures. In §5, we apply the methods to two real-world datasets (a third dataset is shown in electronic supplementary material, E). Section 6 summarizes our main results and offers directions for future work.

The code for our proposed methods and the implementation for bow-tie structure (provided by the authors of [26]) are available at https://github.com/alan-turing-institute/directedCorePeripheryPaper.

## 2. Core–periphery structure

We encode the edges of an *n*-node network in an adjacency matrix **A** = (*A*_*u*,*v*_)_*u*,*v*=1,…,*n*_, with entry *A*_*u*,*v*_ = 1 when there is an edge from node *u* to node *v*, and *A*_*u*,*v*_ = 0 otherwise. We partition the set of nodes into core and periphery sets, resulting in a block partition of the adjacency matrix and a corresponding block probability matrix. In the remainder of the paper, we use the term ‘set’ for members of a node partition and ‘block’ for the partition of a matrix. We shall define a random network model on *n* nodes partitioned into *k* blocks via a *k* × *k* probability matrix **M**, whose entries *M*_*ij*_ give the probability of an edge from a node in block *i* to a node in block *j*, independently of all other edges.

### (a) Core–periphery in undirected networks

The most well-known quantitative formulation of core–periphery structure in undirected networks was introduced by Borgatti & Everett [5]; they propose both a discrete and a continuous model for core–periphery structure. In the discrete notion of core–periphery structure, Borgatti & Everett [5] suggest that an ideal core–periphery structure should consist of a partition of the node set into two non-overlapping sets: a densely connected core and a loosely connected periphery, with dense connections between the core and the periphery. The probability matrix of a network with the idealized core–periphery structure in [5] and the corresponding network-partition representation are given in (2.1),
2.1
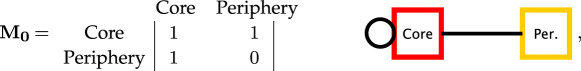

where the network-partition representation on the right-hand side shows edges within and between core and periphery sets. In adjacency matrices of real-world datasets, any structure of the form equation (2.1), if present, is likely to be observed with random noise perturbations.

### (b) Core–periphery structure in directed networks

We now introduce a block model for directed core–periphery structure where the definitions of the core and periphery sets are edge-direction dependent. Starting from equation (2.1), a natural extension to the directed case is to split each of the sets into one that only has incoming edges and another that only has outgoing edges. This yields four sets, which we denote $C_{\text{in}}$ (*core-in*), $C_{\text{out}}$ (*core-out*), $P_{\text{in}}$ (*periphery-in*) and $P_{\text{out}}$ (*periphery-out*), with respective sizes $n_{P_{\text{out}}}$, $n_{C_{\text{in}}}$, $n_{P_{\text{in}}}$ and $n_{C_{\text{out}}}$. We assume that edges do not exist between the periphery sets, and thus that every edge is incident to at least one node in a core set. Respecting edge direction, we place edges between *core-out* and all ‘in’ sets, and between each ‘out’ set and *core-in*. As in equation (2.1), the two core sets are fully internally connected, and the two periphery sets have no internal edges. There are no multiple edges, but self-loops are permitted. The probability matrix and corresponding network partition are given in (2.2),
2.2
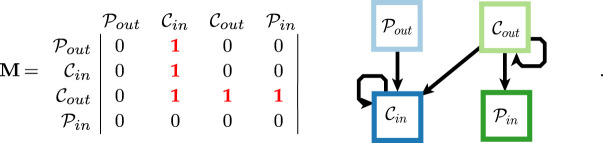

We refer to the structure in **M** as an ‘L’-shaped structure. There are other directed core–periphery structures that one can pursue. In electronic supplementary material, A, we provide a framework of which equation (2.2) is one example, and a block model formulation of bow-tie structure is another example. The particular formulation of the well-known bow-tie structure that falls within our framework is the directed core–periphery structure equation (2.3), where only periphery sets have a definition that is edge-direction dependent, and where we assume that the core and peripheries form a hard partition [22],
2.3
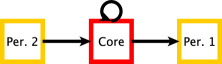

In general, bow-tie can allocate nodes to several sets: there is a core set, an incoming periphery set, an outgoing periphery set and four additional sets corresponding to other connection patterns. There are several known real-world applications of bow-tie structure, such as the Internet [20] and biological networks [27]. We note that the structure in equation (2.2) is not a mere extension of the bow-tie structure as, in contrast to bow-tie, the flow is not uni-directional.

We motivate the structure in equation (2.2) with a few examples. Consider networks that represent a type of information flow, with two sets that receive information ($C_{\text{in}}$ and $P_{\text{in}}$) and two sets that send information ($C_{\text{out}}$ and $P_{\text{out}}$). Furthermore, within each of these categories, there is one set with core-like properties and another set with periphery-like properties. Inspired by Beguerisse-Díaz *et al.* [3], in a Twitter network for example, $C_{\text{in}}$ and $P_{\text{in}}$ could correspond to consumers of information, with $C_{\text{in}}$ having the added property of being a close-knit community that has internal discussions (e.g. interest groups) rather than individuals collecting information independently (e.g. an average user). The sets $C_{\text{out}}$ and $P_{\text{out}}$ could correspond to transmitters of information, with $C_{\text{out}}$ having the added property of being a well-known close-knit community (e.g. broadcasters) rather than individuals spreading information independently (e.g. celebrities). Another class of examples is networks that represent a type of social flux, when there are two sets that entities move out of and two sets that entities move towards. Furthermore, within each of these categories, there is one with core-like properties and one with periphery-like properties. For example, in a faculty hiring network of institutions, $C_{\text{out}}$ may correspond to highly ranked institutions with sought-after alumni, while $C_{\text{in}}$ may correspond to highly sought-after institutions which take in more faculty than they award PhD degrees. For the periphery sets, $P_{\text{out}}$ may correspond to lower-ranked institutions that have placed some faculty in the core but do not attract faculty from higher-ranked institutions, and $P_{\text{in}}$ may correspond to a set of institutions that attract many alumni from highly ranked ones. These ideas will be showcased on real-world data in §5, where we also illustrate that the structure in equation (2.2) yields insights that are not captured by the bow-tie structure.

### (c) Synthetic model for directed core–periphery structure

We now describe a stochastic block model that will be used as a synthetic graph model to benchmark our methods. For any two nodes *u*, *v*, let *X*(*u*, *v*) denote the random variable which equals 1 if there is an edge from *u* to *v*, and 0 otherwise. We refer to *X*(*u*, *v*) as an edge indicator. For an edge indicator which should equal 1 according to the idealized structure (equation (2.2)), let *p*_1_ be the probability that an edge is observed. Similarly for an edge indicator which should be 0 according to the perfect structure (equation (2.2)), let *p*_2_ be the probability that an edge is observed. Interpreting *p*_1_ as *signal* and *p*_2_ as *noise*, we assume that *p*_1_ > *p*_2_ so that the noise does not overwhelm the true structure in equation (2.2). We represent this model as a stochastic block model, denoted by DCP(*p*_1_, *p*_2_), which has independent edges with block probability matrix
2.4
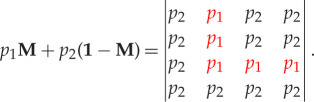

Setting *p*_1_ = 1 and *p*_2_ = 0 recovers the idealized block structure in equation (2.2). The ‘L’-shaped structure in equation (2.4) defines a partition of a network into two cores and two peripheries (see equation (2.2) for the idealized case DCP(1, 0)). We refer to this partition as a ‘planted partition’ throughout the paper. The DCP(*p*_1_, *p*_2_) model allows one to increase the difficulty of the detection by reducing the difference between *p*_1_ and *p*_2_, and to independently modify the expected density of edges matching (respectively, not matching) the planted partition by varying *p*_1_ (respectively, *p*_2_). A case of particular interest is when only the difference between *p*_1_ and *p*_2_ is varied; this is the DCP(1/2 + *p*, 1/2 − *p*) model, where *p* ∈ [0, 0.5]. This model yields the idealized block structure in equation (2.2) when *p* = 0.5, and an Erdős–Rényi (ER) random graph when *p* = 0.

**Figure 1.**
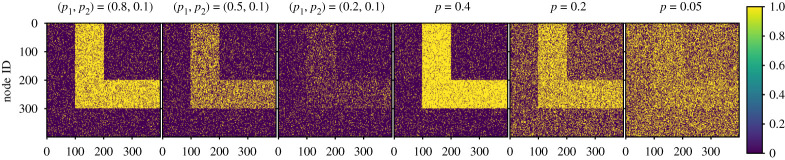
Heatmaps illustrating our model. We present heatmaps of the original adjacency matrix, with *n* = 400 nodes. We generate the first three adjacency matrices with DCP(*p*_1_, *p*_2_) and the next three adjacency matrices with DCP(1/2 + *p*, 1/2 − *p*). Blocks are equally sized in both cases. (Online version in colour.)

Figure 1 displays example adjacency matrices obtained from equation (2.4), with *n* = 400 and equally sized sets $n_{P_{\text{out}}}=n_{C_{\text{in}}}=n_{C_{\text{out}}}=n_{P_{\text{in}}}=100$. In the first three panels, *p*_2_ = 0.1 and *p*_1_ varies. As *p*_1_ decreases with fixed *p*_2_, the ‘L’-shaped structure starts to fade away and the network becomes sparser. The last three panels show realizations of DCP(1/2 + *p*, 1/2 − *p*) adjacency matrices for *p* ∈ {0.4, 0.2, 0.05}, *n* = 400 and four equally sized sets. The ‘L’-shaped structure is less clear for smaller values of *p*.

### (d) Measures of statistical significance and partition quality

In empirical networks, there is often no access to ground truth. It is thus crucial to determine whether a detected partition is simply the result of random chance and does not constitute a meaningful division of a network. Furthermore, different detection methods can produce very different partitions (e.g. by making an implicit trade-off between block size and edge density), and it can be very helpful in practice to have a systematic approach for choosing between methods according to specific criteria of ‘partition quality’. As criteria of partition quality, we employ a *p*-value arising from a Monte Carlo test and an adaptation of the modularity quality function of a partition (see for example eqn (7.58) in [1]).

The *p*-value is given by a Monte Carlo test to assess whether the detected structure could plausibly be explained as arising from random chance, modelled either by a directed ER model without self-loops or by a directed configuration model as in [28]. The test statistic is the difference between the probability of connection within the ‘L’-structure and that outside the ‘L’-structure, i.e.

$$\frac{\sum_{u,v=1}^{n}M_{g_{u},g_{v}}A_{uv}}{\sum_{u,v=1}^{n}M_{g_{u},g_{v}}}-\frac{\sum_{u,v=1}^{n}(1-M_{g_{u},g_{v}})A_{uv}}{\sum_{u,v=1}^{n}(1-M_{g_{u},g_{v}})},$$

where **M** is as in equation (2.2) and *g*_*u*_ is the set assigned to node *u*. To directly measure partition quality, we extend the *core–periphery modularity* measure from [4,29] by replacing the block and community indicators with indicators that match the ‘L’-structure, i.e.

2.5 $$\text{DCPM}(g)=\frac{1}{m}\sum_{u=1}^{n}\sum_{v=1}^{n}(A_{uv}-\langle A\rangle )M_{g_{u}g_{v}},$$

where *m* is the number of edges (with bi-directional edges counted twice) and 〈**A**〉 = *m*/*n*^2^. We call this measure *directed core–periphery modularity* (DCPM). DCPM lies in the range of ( − 1, 1). If there is only one block, then DCPM = 0. If the ‘L’-structure is achieved perfectly, then the number of edges is $m=n_{P_{\text{out}}}n_{C_{\text{in}}}+{\left(n_{C_{\text{in}}}\right)}^{2}+n_{C_{\text{out}}}n_{C_{\text{in}}}+{\left(n_{C_{\text{out}}}\right)}^{2}+n_{C_{\text{out}}}n_{P_{\text{in}}}$ and $\text{DCPM}=1-(1/n^{2})(n_{P_{\text{out}}}n_{C_{\text{in}}}+n_{C_{\text{out}}}^{2}+n_{C_{\text{out}}}n_{C_{\text{in}}}+n_{C_{\text{in}}}^{2}+n_{P_{\text{out}}}n_{C_{\text{in}}})=1-(m/n^{2})$. If, instead, all edges not on the ‘L’ are present, then $\text{DCPM}=-(n_{P_{\text{out}}}n_{C_{\text{in}}}+n_{C_{\text{out}}}^{2}+n_{C_{\text{out}}}n_{C_{\text{in}}}+n_{C_{\text{in}}}^{2}+n_{P_{\text{out}}}n_{C_{\text{in}}})/n^{2}$. DCPM is related to the general form core–periphery quality function introduced in [10].

We note that, in equation (2.5), the null model we compare the observed network against is the expected adjacency matrix under an ER null model, where each edge is generated with the same probability *m*/*n*^2^, independently of all other potential edges, and the expected number of edges is equal to *m*, the observed number of edges. Such a null model was used in [4] to derive a quality function for detecting multiple core–periphery pairs in undirected networks. As high-degree nodes tend to end up in core sets, and low-degree nodes in periphery sets (see for example figure 4 in this paper), using a null model that controls for node degree directly in the quality function can mask a lot of the underlying core–periphery structure [4,18,29]. To circumvent this issue, the authors in [29] modify the core–periphery block structure definition by incorporating an additional block that is different from the core block and its corresponding periphery block. For the purpose of this paper, we use an ER null model and leave the exploration of further null models to future work.

For networks with ground truth (e.g. synthetic networks with planted structure), the accuracy of a partition is measured by the adjusted Rand index (ARI) [30] between the output partition of a method and the ground truth, using the implementation from [31]. The ARI takes values in [ − 1, 1], with 1 indicating a perfect match, and an expected score of approximately 0 under a given model of randomness. A negative value indicates that the agreement between two partitions is less than what is expected from a random labelling. In electronic supplementary material, D(a), we give a detailed description of the ARI, and also consider the alternative similarity measures VOI (variation of information [32]) and NMI (normalized mutual information [33]).

## 3. Core–periphery detection in directed networks

Several challenges arise when considering directed graphs, which makes the immediate extension of existing algorithms from the undirected case difficult. As the adjacency matrix of a directed graph is no longer symmetric, the spectrum becomes complex-valued. Graph clustering methods which have been proposed to handle directed graphs often consider a symmetrized version of the adjacency matrix, such as SaPa [34]. However, certain structural properties of the network may be lost during the symmetrization process, which provides motivation for the development of new methods. In this section, we describe three methods for detecting this novel structure. We pay particular attention to scalability, a crucial consideration in empirical networks, and order the methods by run time, from fast to slow. The first two methods are based on an adaptation of the popular HITS algorithm [25], and the third method is based on likelihood maximization.

### (a) The Hyperlink-Induced Topic Search algorithm

Our first method builds on a well-known algorithm in link analysis known as Hyperlink-Induced Topic Search (HITS) [25]. The HITS algorithm was originally designed to measure the importance of webpages using the structure of directed links between the webpages [35]; authoritative webpages on a topic should not only have large in-degrees (i.e. they constitute hyperlinks on many webpages) but also considerably overlap in the sets of pages that point to them. Referring to authoritative webpages for a topic as ‘authorities’ and to pages that link to many related authorities as ‘hubs’, it follows that a good hub points to many good authorities, and that a good authority is pointed to by many good hubs. The HITS algorithm assigns two scores to each of the *n* nodes, yielding an *n*-dimensional vector **a** of ‘authority scores’ and an *n*-dimensional vector **h** of ‘hub scores’, with **a** = **A**^T^**h** and **h** = **Aa**.

To each node, we assign core and periphery scores based on the HITS algorithm, which we then cluster to obtain a hard partition; we call this the Hits method. Appealing features of the Hits algorithm include (i) it is highly scalable; (ii) it can be adapted to weighted networks; and (iii) it offers some theoretical guarantees on the convergence of the iterative algorithm [25].

**Algorithm for** **Hits**

(i) Initialization: **a** = **h** = **1**_*n*_. Alternate between the following two steps: (a) update **a** = **A**^T^**h**; (b) update **h** = **Aa** . Stop when the change in updates is lower than a pre-defined threshold.
(ii) Normalize **a** and **h** to become unit vectors in some norm [35].
(iii) Compute the *n* × 4 score matrix $S^{\mathrm{HITS}}=[P_{\text{out}}^{\mathrm{HITS}},C_{\text{in}}^{\mathrm{HITS}},C_{\text{out}}^{\mathrm{HITS}},P_{\text{in}}^{\mathrm{HITS}}]$ using the node scores
  3.1 $$\begin{array}{r} C_{\text{in}}^{\mathrm{HITS}}(u)=h(u),\;P_{\text{in}}^{\mathrm{HITS}}(u)={\text{max}}_{v}(C_{\text{out}}^{\mathrm{HITS}}(v))-C_{\text{out}}^{\mathrm{HITS}}(u), \end{array}$$
  3.2 $$\begin{array}{r} C_{\text{out}}^{\mathrm{HITS}}(u)=a(u),\;P_{\text{out}}^{\mathrm{HITS}}(u)={\text{max}}_{v}(C_{\text{in}}^{\mathrm{HITS}}(v))-C_{\text{in}}^{\mathrm{HITS}}(u). \end{array}$$
(iv) Normalize $S^{\mathrm{HITS}}$ so that each row has an *L*_2_-norm of 1 and apply k-means++ to partition the node set into four clusters.
(v) Assign each of the clusters to a set based on the likelihood of each assignment under our stochastic block model formulation (see §2).

**Remark 3.1.**

(i) To motivate the scores equations (3.1) and (3.2), a node should have a high authority score if it has many incoming edges, whereas it would have a high hub score if it has many outgoing edges. Based on the idealized block structure in equation (2.2), nodes with the highest authority scores should also have a high ${\text{C}}_{\text{in}}^{\mathrm{HITS}}$ score, and nodes with the highest hub scores should also have a high ${\text{C}}_{\text{out}}^{\mathrm{HITS}}$ score.
(ii) For step (i) of the algorithm, we use the implementation from NetworkX [36], which computes the hub and authority scores using the leading eigenvector of **A**^T^**A**. As [25] proved that the scores converge to the principal left and right singular vectors of **A**, provided that the initial vectors are not orthogonal to the principal eigenvectors of **A**^T^**A** and **AA**^T^, this is a valid approach.
(iii) Using the same connection between the HITS algorithm and singular value decomposition (SVD) from Kleinberg [37], our scores based on the HITS algorithm can be construed as a variant of the low-rank method in [12], in which we only consider a rank-1 approximation and use the SVD components directly.
(iv) A scoring variant is explored in electronic supplementary material, B, with equations (3.2) and (3.1) performing best on our benchmarks.
(v) Intuitively, the row normalization of **S**^HITS^ from step (iv) allows the rows of **S**^HITS^ (vectors in four-dimensional space) not only to concentrate in four different directions but also to concentrate in a spatial sense and have a small within-set Euclidean distance [38,39].
(vi) Using k-means++ [40] alleviates the issues of unstable clusterings retrieved by k-means [41].

### (b) The Advanced Hits method

We now modify the Hits algorithm such that it considers four distinct scores (rather than two core scores, from which we then compute the periphery scores); we call the resulting method the *Advanced Hits* method, and abbreviate the corresponding algorithm as AdvHits. We do this by incorporating information about the idealized block structure into the algorithm (which, as we show in §4, yields better results on synthetic networks). Namely, instead of using hub and authority scores, in each set, we reward a node for having edge indicators that match the structure in equation (2.2) and penalize otherwise, through the reward–penalty matrix associated with **M**, given by

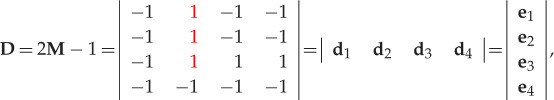

where **d_i_** is the *i*th column vector of **D** and **e_j_** is the *j*th row vector of **D**. The first column/row corresponds to $P_{\text{out}}$, the second column/row to $C_{\text{in}}$, and so on. We use the matrix **D** to define the AdvHits algorithm, with steps detailed below.

**Algorithm for** **AdvHits**

(i) Initialization:
  $${\text{S}}^{\text{Raw}}=[{\text{S}}_{1}^{\text{Raw}},{\text{S}}_{2}^{\text{Raw}},{\text{S}}_{3}^{\text{Raw}},{\text{S}}_{4}^{\text{Raw}}]=[{\text{P}}_{\text{out}}^{\text{Raw}},{\text{C}}_{\text{in}}^{\text{Raw}},{\text{C}}_{\text{out}}^{\text{Raw}},{\text{P}}_{\text{in}}^{\text{Raw}}]=U_{n},$$
  where **U**_*n*_ is an *n* × 4 matrix of independently drawn uniform (0, 1) random variables.
(ii) For nodes *u* ∈ {1, …, *n*} let $B(u)=\min\{P_{\text{out}}^{\text{Raw}}(u),C_{\text{in}}^{\text{Raw}}(u),C_{\text{out}}^{\text{Raw}}(u),P_{\text{in}}^{\text{Raw}}(u)\}$, and calculate, for sets *i* ∈ {1, 2, 3, 4},
  3.3 $$S_{i}^{\text{Nrm}}(u)=\frac{S_{i}^{\text{Raw}}(u)-B(u)}{\sum_{k=1}^{4}(S_{k}^{\text{Raw}}(u)-B(u))}.$$
  If for a node *u*, the raw scores for each set are equal, up to floating point error (defined as the denominator of equation (3.3) being less than 10^−10^), this implies an equal affinity to each set and thus we set $S_{i}^{\text{Nrm}}(j)=0.25$.
(iii) For *i* ∈ {1, …, 4}:
  (a) Update $S_{i}^{\text{Raw}}$:
    3.4 $$\begin{array}{rl} S_{i}^{\text{Raw}} & =\left(1-\frac{m}{n^{2}}\right)AS^{\text{Nrm}}{\text{e}}_{i}^{\text{T}}+\frac{m}{n^{2}}(1-A)S^{\text{Nrm}}(-{\text{e}}_{i}^{\text{T}})\\ & \;+\left(1-\frac{m}{n^{2}}\right)A^{\text{T}}S^{\text{Nrm}}{\text{d}}_{i}+\frac{m}{n^{2}}(1-A^{\text{T}})S^{\text{Nrm}}(-{\text{d}}_{i}). \end{array}$$
  (b) Recompute *S*^Nrm^ using the procedure in step (ii).
  (c) Measure and record the change in $S_{i}^{\text{Nrm}}$.
(iv) If the largest change observed in $S_{i}^{\text{Nrm}}$ is greater than 10^−8^, return to step (iii).
(v) Apply k-means++ to **S**^Nrm^ to partition the node set into four clusters.
(vi) Assign each of the clusters to a set based on the likelihood of each assignment under our stochastic block model formulation (see §2).

**Remark 3.2.**

(i) The first term in equation (3.4) rewards/penalizes the outgoing edges, the second the missing outgoing edges, the third the incoming edges, and the fourth the missing incoming edges. The multiplicative constants are chosen to weigh edges in each direction evenly, and to fix the contribution of non-edges to be equal to that of edges.
(ii) We envision the score to represent the affinity of a given node to each set. Thus, the normalization step is included so that the scores of an individual node sum to 1. We include *B*(*u*) as the scores in equation (3.4) can be negative and thus we shift the values to be all positive (and rescale).
(iii) The general iteration can fail to converge within 1000 iterations. If the scheme has not converged after 1000 steps, we fall back to a scheme which updates the scores on each node in turn, which often empirically removes the convergence issue with the cost of additional computational complexity.

### (c) Likelihood maximization

Our third proposed method, MaxLike, maximizes the likelihood of the directed core–periphery model equation (2.4), which is a stochastic block model with four blocks and our particular connection structure. To maximize the likelihood numerically, we use a procedure from [42], which we call MaxLike; this procedure updates the set assignment of the node that maximally increases/minimally decreases the likelihood at each step, and then repeats the procedure with the remaining non-updated nodes. The complete algorithm is given in electronic supplementary material, C. For multimodal or shallow likelihood surfaces, the maximum-likelihood algorithms may fail to detect the maximum, and instead find a local optimum. To alleviate this concern, we use a range of initial values for the algorithms.

In our preliminary analysis, we also employed a related faster, greedy likelihood maximization algorithm. We found that MaxLike slightly outperformed the faster approach on accuracy, and hence do not present the fast greedy method here.

## 4. Numerical experiments on synthetic data

In order to compare the performance of the methods from §3, we create three benchmarks using the synthetic model DCP(*p*_1_, *p*_2_) from §2. Leveraging the fact that we have access to a ground truth partition (here, a planted partition), the purpose of these benchmarks is (i) to compare our approaches with other methods from the literature and (ii) to assess the effectiveness of the *p*-value and the DCPM as indicators of core–periphery structure. We also use the benchmark to assess the run time of the algorithms. For the methods comparison, we compare Hits, AdvHits and MaxLike with a naive classifier (Deg.), which performs k-means++ [40], clustering solely on the in- and out-degree of each node. We also compare them against two well-known fast approaches for directed networks, namely SaPa from [34] and DiSum from [43]; implementation details and variants can be found in electronic supplementary material, D. For brevity, we only include the best-performing SaPa and DiSum variant, namely SaPa2, using degree-discounted symmetrization, and DiSum3, a combined row and column clustering into four sets, using the concatenation of the left and right singular vectors. Both SaPa and DiSum perform degree normalization, which may limit their performance. Moreover, our methods are compared against the stochastic block modelling fitting approach GraphTool [44], based on [2,45], which minimizes the minimum description length of the observed data. To make this a fair comparison, we do not use a degree-corrected block model but instead a standard stochastic block model, and we fix the number of sets at four.

The second goal is to assess on synthetic networks whether our ranking of method performance based on *p*-value and DCPM is qualitatively robust across measures that do not require knowledge of a ground truth partition. To this end, we compare these rankings with those obtained with measures that do leverage ground truth, namely the ARI.

### (a) Results for the benchmark networks

#### (i) Benchmark 1

We test our approaches using our 1-parameter SBM DCP(1/2 + *p*, 1/2 − *p*), with equally sized sets, and varying p∈{0.5,0.49,0.48,…,0.21}∪{0.195,0.19,0.185,…,0.005}, the finer discretization step zooming in on the parameter regime which corresponds to the planted partition being weak. We average over 50 network samples for each value of *p*. Recall that for *p* = 0.5 the planted partition corresponds to the idealized block structure in equation (2.2) and for *p* = 0 the planted partition corresponds to an ER random graph with edge probability 0.5.

The performance results for sets of size 100 (*n* = 400) are shown in table 1, giving the ARI for *p* = 0.4 and for values of *p* between 0.1 and 0.02 with step size 0.01, in decreasing order (with results for the full parameter sweep in electronic supplementary material, D). With regards to ARI, MaxLike performs best for *p* in the range of 0.1–0.03, with performance deteriorating when the noise approaches the signal. Above a certain threshold of *p* (roughly around *p* = 0.25, results shown in electronic supplementary material, D, figure SI 1I), many approaches, including the degree-based one Deg., achieve optimal performance, indicating that, in this region of the networks obtained with benchmark 1, the degrees alone are sufficient to uncover the structure. For NMI and VOI, we observe similar qualitative results; see electronic supplementary material, D.

**Table 1.** Average ARI of the methods under comparison on benchmark 1 (DCP(1/2 + *p*, 1/2 − *p*)) for different values of *p*, and with network size *n* = 400. The largest values for each column are given in italics.

*p*	0.4	0.1	0.09	0.08	0.07
Deg	*1.0*	0.878	0.819	0.753	0.663
Disum	0.995	0.383	0.277	0.193	0.117
SaPa	*1.0*	0.405	0.276	0.202	0.144
GraphTool	*1.0*	0.996	0.985	0.968	0.921
Hits	*1.0*	0.909	0.852	0.78	0.692
AdvHits	*1.0*	0.972	0.946	0.901	0.814
MaxLike	*1.0*	*0.997*	*0.986*	*0.971*	*0.931*

*p*	0.06	0.05	0.04	0.03	0.02
Deg	0.536	0.408	0.281	0.163	0.0767
Disum	0.0506	0.0171	0.00651	0.0021	0.000614
SaPa	0.0811	0.0306	0.00809	0.00274	0.00085
GraphTool	0.655	0.0104	0.000119	2.08 × 10^−05^	2.73 × 10^−05^
Hits	0.562	0.423	0.275	0.152	0.071
AdvHits	0.693	0.525	0.333	0.168	*0.0777*
MaxLike	*0.831*	*0.675*	*0.42*	*0.195*	0.0577

The performance of GraphTool collapses as *p* gets close to 0 (similar behaviour is observed for *n* = 1000; see electronic supplementary material, D). Further investigation indicated that, for low values of *p*, GraphTool often places most nodes in a single set (see electronic supplementary material, D for further details).

Benchmark 1 is also used to assess the run time of the algorithms. The slowest of our methods across all values of *p* is MaxLike. For small *p*, Hits is the fastest of our methods, whereas for larger *p* it can be overtaken by AdvHits; both are faster than GraphTool. Within methods, the performance is relatively constant for Hits, while it speeds up for decreasing *p* in AdvHits and MaxLike. The detailed results can be found in electronic supplementary material, D.

#### (ii) Benchmark 2

We use the model DCP(*p*_1_, *p*_2_), again with all four sets of the same size *n*/4. In this model, the edge probabilities (*p*_1_, *p*_2_) vary the density and the strength of the core–periphery structure independently. To this end, we vary *p*_1_ and the ratio 0 ≤ *p*_2_/*p*_1_ < 1. For a given *p*_1_, *p*_2_/*p*_1_ = 0 corresponds to the strongest structure and *p*_2_/*p*_1_ = 1 to the weakest structure. We generate 50 networks each with *p*_1_ ∈ {0.025, 0.05, …, 1.0} and *p*_2_/*p*_1_ ∈ {0, 0.05, …, 0.95}, resulting in 820 parameter instances of (*p*_1_, *p*_2_/*p*_1_). The contours corresponding to an average ARI of 0.75 and an average ARI of 0.9 for *n* = 400 and *n* = 1000 are shown in electronic supplementary material, D.

Similar to the situation in benchmark 1, the full-likelihood approach MaxLike outperforms all other methods, with GraphTool also performing well and the performance of AdvHits coming close and outperforming GraphTool in certain regions.

#### (iii) Benchmark 3

Benchmark 3 assesses the sensitivity of our methods to different set sizes. We use the model DCP(1/2 + *p*, 1/2 − *p*). We fix *p* = 0.1, as we observed in table 1 that this value is sufficiently small to highlight variation in performance between our approaches but sufficiently large that most of the methods can detect the underlying structure. We then consider the effect of size variation for each set in turn, by fixing the size of the remaining three sets. For example, to vary the size of $P_{\text{out}}$, we fix $n_{C_{\text{in}}}=n_{C_{\text{out}}}=n_{P_{\text{in}}}=n_{1}$ and test performance when we let $n_{P_{\text{out}}}=n_{2}\in \{2^{-3}n_{1},2^{-2}n_{1},\ldots ,2^{3}n_{1}\}$, with equivalent formulations for the other sets. Thus for *n*_2_/*n*_1_ = 1 we have equal-sized sets, which is equivalent to the model in benchmark 1; for *n*_2_/*n*_1_ > 1 one set is larger than the remaining sets; and for *n*_2_/*n*_1_ < 1 one set is smaller than the others.

Results are shown in electronic supplementary material, D for *n*_1_ = 100 (*n*_2_/*n*_1_ = 1 implies a 400-node network). MaxLike slightly outperforms GraphTool, and is the overall best performer, appearing to be robust to set size changes. AdvHits usually outperforms the other approaches; however, for larger sets, the AdvHits is in some cases even outperformed by Deg.

### (b) Performance of the *p*-value and DCPM to capture ground truth

To investigate whether the *p*-values and DCPM introduced in §2 are appropriate to assess partition quality, we test the relationship between our proposed quality measures and ARI on a set of benchmark networks. We create these networks using the synthetic model for benchmark 1, i.e. DCP(1/2 + *p*, 1/2 − *p*), with three values of *p* focusing on the region where the planted partition is detectable (*p* = 0.1); marginally detectable (*p* = 0.04); and (mostly) undetectable (*p* = 0.02). We note that, for large *p*, all of the methods will be able to uncover the exact partition and thus each partition would have an ARI of 1 (table 1), with differences in DCPM driven by the strength of the embedded structure. For computational reasons, we restrict the experiment to 20 networks for each *p*, and use 250 null replicates for each Monte Carlo test. Each of our three methods is applied to each network, and thus each network gives rise to three *p*-values and DCPM values.

**Figure 2.**
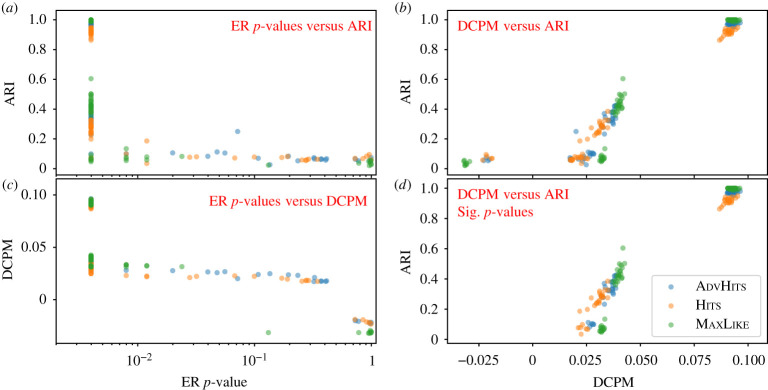
Scatter plots for *p*-value, DCPM and ARI, using the partitions given by each of our methods on networks taken from DCP(1/2 + *p*, 1/2 − *p*) with *p* ∈ [0.015, 0.04, 0.1], with 20 networks for each *p*. (*a*) ER model *p*-value against ARI. (*b*) DCPM against ARI. (*c*) ER model *p*-value against DCPM. (*d*) ARI against DCPM using only networks that are significant (*p*-value<0.05) in both the ER model and the configuration model test. The colour of each of the points represents the method used. (Online version in colour.)

For good partitions, the ARI should be high, the *p*-value should be low and the DCPM value should be high. Hence ARI and the *p*-value should be negatively correlated, the *p*-value and DCPM should be negatively correlated, and ARI and DCPM should be positively correlated. For robustness, we assess correlation by Kendall’s *τ* rank correlation coefficient. For both the ER and configuration mode *p*-values, we observe a moderate negative correlation with ARI (ER: −0.599, configuration: −0.506; data for the configuration model not shown). The correlation between DCPM and ER *p*-value is −0.655, and the correlation between DCPM and ARI is 0.774. Figure 2*a* illustrates that selecting partitions with an ER *p*-value less than 0.05 is successful at filtering out partitions with a low ARI, but struggles to separate partitions with mid-range ARI from networks with high ARI. Focusing only on network partitions with a *p*-value of less than 0.05 in both the ER and the configuration model test, as shown in figure 2*d*, we note that DCPM further differentiates the partitions with low *p*-value and gives a correlation of 0.774 with ARI. The directions of all of these correlations are as expected. If the observations were independent, then these correlations would be highly statistically significant. Thus, while not conclusive evidence, the level of correlation supports the use of our *p*-value test and DCPM to identify partitions.

As further support for this claim, table 2 presents the average ER and configuration *p*-value, average DCPM values and average ARI, broken down by method and model parameter. As expected for good partitions, we observe low *p*-values for strong structures (*p* = 0.1, ARI>0.9), higher *p*-values for weaker structures (*p* = 0.04, 0.25 < ARI < 0.45) and non-significant *p*-values for very weak or non-existent structures (*p* = 0.02, ARI < 0.1).^1^ In particular, whenever average ARI ≥0.4 in table 2, all *p*-values are significant. Thus, we find that both the *p*-value and the DCPM can be used as a proxy for the ARI, displaying a moderate correlation. The DCPM is particularly useful to extract more detailed information for partitions which exhibit low *p*-values. In particular, table 2 and electronic supplementary material, D indicate that using average DCPM as an approach to rank methods overall yields qualitatively similar results to ARI.

**Table 2.** Average *p*-value (ER and configuration model), DCPM and ARI, over 20 networks, with a breakdown by method and parameter in a DCP(1/2 + *p*, 1/2 − *p*) model; *p*-values are rounded to 3 dp. The corresponding sample standard deviations are shown in electronic supplementary material, D, table S5.

	0.1			
	*p*-value			
*p*	ER	Con.	DCPM	ARI
Hits	0.004	0.004	0.091	0.916
AdvHits	0.004	0.004	0.093	0.974
MaxLike	0.004	0.004	0.093	0.997

	0.04			
	*p*-value			
*p*	ER	Con.	DCPM	ARI
Hits	0.004	0.004	0.031	0.274
AdvHits	0.007	0.008	0.035	0.340
MaxLike	0.004	0.004	0.040	0.439

	0.02			
	*p*-value			
*p*	ER	Con.	DCPM	ARI
Hits	0.325	0.269	0.011	0.071
AdvHits	0.327	0.412	0.014	0.074
MaxLike	0.344	0.4	0.007	0.059

In table 2, MaxLike and AdvHits tend to have the highest average DCPM and ARI. In electronic supplementary material, D, we show that this observation is robust across further values of *p*. Overall, our ranking of method performance based on average partition quality values is thus robust across DCPM and ARI, for different values of *p* in DCP(1/2 + *p*, 1/2 − *p*).

To illuminate the relationship between DCPM and ARI further, for *p* = 0.1 we observe a Kendall correlation of 0.315 between them across methods; for *p* = 0.04 this correlation increases to 0.753, while for *p* = 0.02 the correlation decreases to 0.367 (all rounded to 3 dp). For *p* = 0.1, there is little noise and hence variation in DCPM, ranging between 0.0868 and 0.0964, nor in ARI, ranging from 0.863 to 1; the structure is so strong that much of it is picked up by the methods, and the noise which both methods pick up will be small and a Kendall correlation will mainly relate to this noise. For *p* = 0.04 there is a moderate signal; DCPM ranges between 0.020 and 0.0427 while ARI ranges between 0.0186 and 0.605. Here the strong correlation between DCPM and ARI supports the value of DCPM as a proxy for ARI in choosing partitions which resemble the ground truth. For *p* = 0.02 there is little signal in the data and hence DCPM and ARI will be noisy; DCPM here ranges between −0.032 and 0.033, while ARI ranges between 0.021 and 0.132. Owing to the high level of noise, none of the methods will tend to give very good partitions, and the correlation between the measures will be relatively weak. Notably, in all cases the correlation is larger than 0.3, revealing a moderate correlation across the range.

### (c) Procedure

Our procedure to select between methods and partitions in a systematic manner is as follows.

**Procedure:**

(i) Compute partitions using each computationally tractable method.
(ii) For each partition, use our Monte Carlo test to see if it deviates from random, with respect both to ER and to the directed configuration model, and exclude the partitions that are not significant.
(iii) Rank the selected significant partitions for further analysis using DCPM.

## 5. Application to real-world data

In this section, we apply our methods to three real-world datasets, namely faculty hiring data (*Faculty*) from [46] (§5a), trade data (*Trade*) from [47] (§5b) and political blogs (*Blogs*) from [48] (presented in the electronic supplementary material, F for brevity). In each case, our methods find a division into four sets, and we explore the identified structure using known underlying attributes. We use the procedure which we validated on synthetic data in §4, using DCPM to only rank partitions with significant *p*-values. We also assess the consistency of the partitions, both within and across each of the approaches, by computing the within-method ARI between the resultant partitions and the ARI between methods of different types.

Moreover, we compare the partitions with the structure uncovered by bow-tie [20], as discussed in §2. As bow-tie allocates nodes to seven sets, we consider the ARI between the partition into seven sets (BowTie) and the partition induced only by the core set and the in- and out-periphery sets (BowTieAdj). When computing the ARI between the partition given by BowTieAdj with another partition S, we consider the partition induced by S on the node set in BowTieAdj (by construction, the ARI between BowTieAdj and BowTie is always 1).

**Figure 3.**
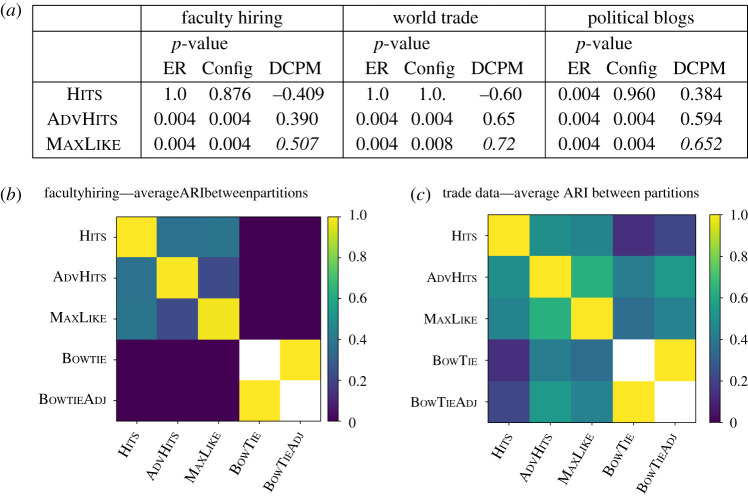
(*a*) Performance of the methods on each of the real-world datasets. The *p*-values are computed using our Monte Carlo test with 250 samples from the null distribution. The values have been rounded to 3 dp. The largest values of DCPM (from §2) for each dataset are given in italics. (*b,c*) The ARI between the partitions uncovered by each method: (*b*) *Faculty*, (*c*) *Trade*. Negative values are set to 0. For our methods, we compare with 11 runs and show the average similarity between all pairs of partitions, whereas for bow-tie, we use a single run (the algorithm is deterministic) and thus display a blank (white) square on the corresponding diagonal blocks. To compare with bow-tie, we compare both with the partition into seven sets and the BOWTIEADJ partition formed by a subset of the nodes corresponding to the main three sets. (Online version in colour.)

Figure 3*a* shows a summary table for the three real-world datasets; the *p*-values correlate with the DCPM measure on all three datasets, and the value of DCPM is always highest for the likelihood approach. We thus focus our interpretation on the output partition obtained with Maxlike.

### (a) Faculty hiring

In the faculty hiring network from [46], nodes are academic institutions, and a directed edge from institution *u* to *v* indicates that an academic received their PhD at *u* and then became faculty at *v*. The dataset is divided by gender and faculty position, and into three fields (business, computer science and history). For brevity, we only consider the overall connection pattern in computer science. This list includes 23 Canadian institutions in addition to 182 American institutions. The data were collected between May 2011 and March 2012. They include 5032 faculty, of whom 2400 are full professors, 1772 associate professors and 860 assistant professors; 87% of these faculty received doctorates which were granted by institutions within the sampled set. In [46], it is found that a large percentage of the faculty is trained by a small number of institutions, and it is suggested that there exists a core–periphery-like structure in the faculty hiring network.

**Figure 4.**
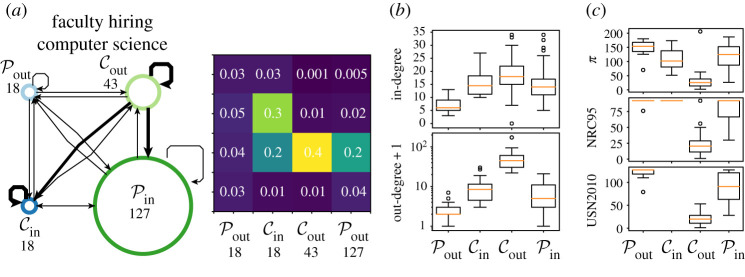
Structures in *Faculty*. Summary network diagram associated with the uncovered structure for MaxLike. The size of each of the nodes is proportional to the number of nodes in the corresponding set, and the width of the lines is given by the percentage of edges that are present between the sets. Partitions in *Faculty*. (*a*) Boxplot of in- and out-degrees in each of the sets in MaxLike. (*b*) Boxplot of in- and out-degrees in each of the sets in AdvHits. To visualize the out-degrees on a log scale, we add 1 to the degrees. (*c*) Boxplot of the ranking in [46], denoted *π*, the ranking in NRC95 and the ranking in USN2010 in each of the sets in MaxLike. If a ranking is not reported for an institution, we exclude the institution from the boxplot. (Online version in colour.)

We apply our procedure to this dataset, and find that the results from the AdvHits variants and the likelihood method MaxLike are significant at 5% under both random null models, whereas the other approaches are not (figure 3). Next, we consider the DCPM score between the significant partitions (figure 3) and note that MaxLike (0.507) yields a stronger structure than AdvHits (0.390), and hence we focus on the MaxLike partition, which is shown in figure 4.

The results in figure 4 show a clear ‘L’-shaped structure, albeit with a weakly defined $P_{\text{out}}$. To interpret these sets, we first compare them against several university rankings. In each of the sets found using MaxLike, figure 4*c* shows the university ranking *π* obtained by Clauset *et al*. [46], and the two other university rankings used in [46], abbreviated NRC95 and USN2010. Here, the NRC95 ranking from 1995 was used because the computer science community rejected the 2010 NRC ranking for computer science as inaccurate. The NRC ranked only a subset of the institutions; all other institutions were assigned the highest NRC rank +1 = 92. The set $C_{\text{out}}$ has considerably smaller ranks than the other sets, indicating that $C_{\text{out}}$ is enriched for highly ranked institutions. Upon inspection, we find that $C_{\text{out}}$ consists of institutions including Harvard, Stanford, Massachusetts Institute of Technology, and also a node that represents institutions outside of the dataset. The set $P_{\text{in}}$ from MaxLike appears to represent a second tier of institutions that take academics from the universities in $C_{\text{out}}$ (figure 4) but do not return them to the job market. This observation can again be validated by considering the rankings in [46] (figure 4*c*). The $C_{\text{in}}$ set loosely fits the expected structure with a strong incoming link from $C_{\text{out}}$ and a strong internal connection (figure 4), suggesting a different role from that of the institutions in $P_{\text{in}}$. A visual inspection of the nodes in $C_{\text{in}}$ reveals that 100% of the institutions in $C_{\text{in}}$ are Canadian (also explaining the lack of ranking in USN2010 (figure 4*c*)). By contrast, the proportion of Canadian universities in $P_{\text{out}}$ is 11.1%, in $C_{\text{out}}$ it is 2.3% and in $P_{\text{in}}$ it is 0.79%. This finding suggests that Canadian universities tend to play a structurally different role from US universities, tending to recruit faculty from other Canadian universities as well as from the top US schools. In [46], the insularity of Canada was already noted, but without a core–periphery interpretation. One possible interpretation of this grouping is salary. In 2012, it was found that Canadian public universities offered a better faculty pay on average than US public universities; see [49].

Finally, $P_{\text{out}}$ is weakly connected both internally and to the remainder of the network and does not strongly match the ‘L’-structure (figure 4). In each of the rankings (figure 4*c*), $P_{\text{out}}$ has slightly lower average ranks than the other sets (with the exception of $C_{\text{in}}$, owing to the default/missing rankings of Canadian institutions). This could indicate that $P_{\text{out}}$ consists of lower ranked institutions which are not strong enough to attract faculty from the larger set of institutions. The in- and out-degree distributions (figure 4*b*) show that $P_{\text{out}}$ has lower in- and out-degree distributions than the other sets. Thus, an alternative hypothesis is that $P_{\text{out}}$ consists of universities with smaller computer science departments which do not interact with the wider network. We leave addressing this interpretation to future work. In either case, the institutions in $P_{\text{out}}$ do not appear to match the pattern observed in the remainder of the network and hence it is plausible to delegate them into one set.

Overall, in this real-world dataset, we demonstrated the power of our method by uncovering an interesting structure that includes a $C_{\text{in}}$ which captures Canadian universities that appear to recruit faculty from top-ranked US institutions, but also recruit from other Canadian institutions in $C_{\text{in}}$.

### (b) World trade

The world trade network from [47] has countries as nodes and directed edges between countries representing trade. For simplicity, we focus on data from the year 2000 and restrict our attention to the trade in ‘armoured fighting vehicles, war firearms, ammunition, parts’ (Standardized International Trade Classification class 9510). We remove trades that do not correspond to a specific country, resulting in a total of 256 trades involving 101 countries, which leads to a network density of approximately 0.025.

Following our procedure, we first consider the *p*-values of our Monte Carlo test. AdvHits and MaxLike show significant deviation from random when compared with the directed ER and directed configuration models (figure 3). When calculating the DCPM for statistically significant partitions, we observe a similar ordering to that of the *Faculty* dataset results, with MaxLike having the highest DCPM (0.72), AdvHits having the second highest DCPM (0.65) and finally Hits with a DCPM of −0.60.

The ARIs in figure 3 show considerable similarity between the MaxLike and AdvHits, with a weaker similarity between Hits and the BowTie variants. Considering the similarity to BowTie, the connected component-based BowTie performs better on this sparser dataset, producing four sizeable sets and two singleton sets (unlike in *Faculty* with two sizeable sets and one singleton set). However, while there is some similarity to our partitions (as demonstrated by a larger value of ARI), the structures captured by each approach are distinct and complementary. For example, focusing on the structure with the highest DCPM (MaxLike), the BowTie ‘core’ combines our $P_{\text{out}}$ and $C_{\text{out}}$, capturing ≈93% of the nodes in $P_{\text{out}}$ (26) and ≈82% of the nodes in $C_{\text{out}}$ (9). Overall, this demonstrates that, in this dataset, BowTie does not distinguish between what we will demonstrate below are two distinct structural roles. Furthermore, BowTie splits our $P_{\text{in}}$ set into two. A similar comparison of the division of the sets holds between BowTie and AdvHits, indicating that the differences between BowTie and the methods to which it is similar in figure 3 are robust.

**Figure 5.**
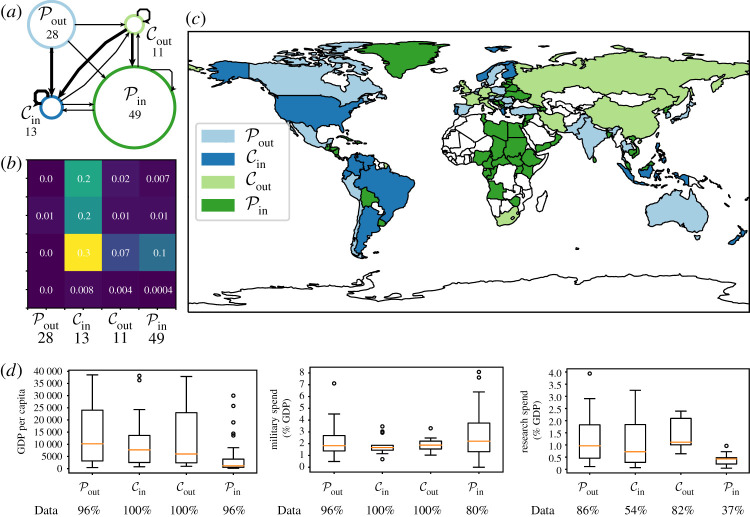
Structures in the *Trade* dataset. We show summary network diagrams associated with the uncovered structures for the MaxLike partition on the *Trade* network, constructed using trades from the category ‘armoured fighting vehicles, war firearms, ammunition, parts’ category from the year 2000. In (*a*), we show a summary of the uncovered structure. The size of each of the nodes is proportional to the number of nodes in each set, and the width of the lines is given by the percentage of edges that are present between the sets. In (*b*), we display the percentage of edges between each pair of blocks, allowing for a visualization of the ‘L’-structure. (*c*) Visualizes the partition on a world map with the colours corresponding to each of the uncovered sets. In (*d*), we display boxplots of three covariates of the uncovered groups, namely *GDP per capita*, *military spend* as a percentage of GDP, and *research spend* as a percentage of GDP. To render the covariates comparable with the partitions from the year 2000, we restrict the covariate data to be from the same year. We note that data from year 2000 are not available for all country covariate pairs, and thus we present the percentage of countries with data in each group in the bottom row of each plot. (Online version in colour.)

Following our procedure, we now focus on the structure with the highest DCPM (MaxLike). It has the ‘L’-shaped structure (figure 5*a*), with smaller core sets and larger periphery sets. To support our interpretation of the structures, we also present summaries of some of their covariates for the year 2000, namely gross domestic product (GDP) per capita research spend, and military spend, the last two as a percentage of GDP. We obtain these covariates from the World Bank with the ‘wbdata’ package [50], using ‘GDP per capita (current US$)’ licensed under CC-BY 4.0 [51], ‘military expenditure (% of GDP)’ from the Stockholm International Peace Research Institute [52] and ‘research and development expenditure (% of GDP)’ from the UNESCO Institute for Statistics and licensed under CC BY-4.0 [53]. Not all country covariate pairs have the covariate data available. For completeness, in figure 5*d*, we report the percentage of data points we have available, split by covariate and group.

From figure 5, key patterns emerge, with $C_{\text{out}}$ consisting of somewhat wealthy countries, with a higher research spend as a percentage of GDP and a high density of export links. This set includes several European countries (France/Monaco, Germany, Italy, UK, Switzerland/Liechtenstein, the Czech Republic and Slovakia), as well as Russia, China, Iran and South Africa.

By contrast, the set $C_{\text{in}}$ has a higher median GDP per capita but with a lower upper quartile and, on average, a lower research spend than $C_{\text{out}}$ (figure 5). It includes several South American countries (Argentina, Brazil, Colombia, Ecuador and Venezuela), several European countries (Greece, Norway and Finland) and several countries in southeast Asia/Oceania (Philippines, Indonesia and New Zealand). A key player in the network appears to be the USA, with a very high in-degree of 45 (the country with the second-largest in-degree is Norway, also in $C_{\text{in}}$, with an in-degree of 15) and a lower out-degree of 14 (11 of which are in $C_{\text{in}}$); the country with the largest out-degree of 16 is the Czech Republic (six of which are in $C_{\text{in}}$). To assess the robustness of this allocation, we removed the USA and all its degree-1 neighbours (a total of four nodes); the resulting core–periphery structure is similar, with nine nodes changing sets.

The set $P_{\text{out}}$ appears to consist of economies which are not large exporters, but support the countries in $C_{\text{in}}$. The group consists of 14 European nations (e.g. Austria, Belgium, The Netherlands and Spain), several nations from Asia (India, Pakistan, Japan, South Korea, Singapore, Taiwan and Thailand), three Latin American countries (Chile, Mexico and Peru) and several additional countries which do not fit into a clear division. Finally, $P_{\text{in}}$ consists of nations that buy from the main exporters, but do not export themselves. This group is large (49 nodes), and includes 17 African nations, representing most of the African nations in the dataset. An additional set of seven nations were either part or closely aligned with the USSR (e.g. Estonia, Latvia, Lithuania and Ukraine). Finally, there is also a group of six Latin American countries and seven Middle Eastern countries, including Syria and Oman. The set $P_{\text{in}}$ appears to have on average lower GDP per capita than other groups (figure 5), with a higher range of military spending as a proportion of GDP. For this group, data on the research spend as a percentage of GDP are only available for 37% of the countries. We observe that, for these countries, it is (on average) much lower than for the other groups.

In conclusion, our procedure uncovers four groups, each with a different structural role in the trade network. We have explored the roles that each of these groups might play in the global market, and while we cannot rule out data quality issues, the partition found does uncover latent strong patterns which we have validated by considering external covariates.

## 6. Conclusion and future work

We provide the first comprehensive treatment of a directed discrete core–periphery structure which is not a simple extension of the bow-tie structure. The structure we introduced consists of two core sets and two periphery sets defined in an edge-direction-dependent way, each with a unique connection profile.

In order to identify when this structure is statistically significant in real-world networks, and to rank partitions uncovered by different methods in a systematic manner, we introduce two quality measures: *p*-values from Monte Carlo tests and a modularity-like measure which we call DCPM. We validate both measures on synthetic benchmarks where ground truth is available.

To detect this structure algorithmically, we propose three methods, Hits, AdvHits and MaxLike, each with a different trade-off between accuracy and scalability, and find that MaxLike tends to outperform AdvHits, as well as the standard methods from the literature against which it was compared.

Using our quality functions to select and prioritize partitions, we explore the existence of our directed core–periphery structure in three real-world datasets, namely a faculty hiring network, a world trade network and a political blog network. In each dataset, we found at least one significant structure when comparing with random ER and configuration model graphs.

(i) In the faculty hiring dataset, the MaxLike partition uncovers a new structure, namely Canadian universities which have a large number of links with the top US universities, but also appear to strongly recruit from their own universities, indicating a complementary structure to the one found in [46].
(ii) In the trade data, we uncover four sets of countries that play a structurally different role in the global arms trade, and we validate this structure using covariate data from the World Bank.
(iii) In the political blogs dataset, we uncover a $C_{\text{in}}$ core, which we hypothesize to consist of authorities that are highly referenced, and a $C_{\text{out}}$ core, which links to a large number of other blogs. We support this hypothesis by noting that $C_{\text{in}}$ has a much lower percentage of ‘blogspot’ sites than the other set, and that $C_{\text{in}}$ contains all but one of the top blogs identified by Adamic & Glance [48].

In cases where one of our methods does not yield a statistically significant partition or yields a partition with a low value of DCPM (e.g. Hits with *Trade*), it can be important to inspect the output partition before disregarding it. We have observed that in certain cases this can occur because the assignment of clusters to the sets $P_{\text{out}}$, $C_{\text{in}}$, $C_{\text{out}}$ and $P_{\text{in}}$ with the highest likelihood in the final step of each method (see §3) has low density within the ‘L’ and high density outside the ‘L’. This phenomenon may occur because the stochastic block model which assigns the group labels of recovered sets rewards homogeneity but does not penalize for sparseness within the ‘L’. One could modify our implementation into a constrained likelihood optimization where one would obtain partitions with potentially lower likelihood but a more pronounced ‘L’ structure.

### (a) Future research directions

There are a number of interesting directions to explore in future work. We start with the specification of the core–periphery structure. The faculty data highlight that some nodes simply may not fit the core–periphery pattern, and thus, following the formulation of bow-tie, it would be interesting to explore modifications to our approaches that would allow for not placing nodes if they do not match the pattern (for example, by introducing a separate set for outlier nodes). As detailed in electronic supplementary material, A, other directed core–periphery patterns are possible. Some of our methods could be adapted to detect such core–periphery patterns. In principle, all possible core–periphery structures could be tested simultaneously, with an appropriate correction for multiple testing. Such a development should of course be motivated by a suitable dataset which allows for interpretation of the results. More generally, meso-scale structures may change over time, and it would be fruitful to extend our structure and methods to include time series of networks.

Next, we propose some future directions regarding the methods for detecting core–periphery structure. The first direction concerns scalability. Depending on the size of the dataset under investigation, a user of our methods may wish to compromise accuracy for scalability (e.g. by using Hits or AdvHits instead of MaxLike). Another scalable method to potentially consider stems from the observation that the expected adjacency matrix (under a suitable directed stochastic block model) is a low-rank matrix. With this in mind, the observed adjacency matrix can be construed as a low-rank perturbation of a random matrix, and, therefore, one could leverage the top singular vectors of the adjacency matrix to propose an algorithm for directed core–periphery detection. The advantage of this approach is that it is amenable to a theoretical analysis and one could provide guarantees on the recovered solution, by using tools from matrix perturbation and random matrix theory. In our preliminary numerical experiments, such an SVD-based approach outperforms the standard methods, and, while outperformed by MaxLike and AdvHits, it is considerable faster. More details can be found in [23]. Further future work could explore graph regularization techniques, which may increase performance for sparse networks. Another direction for future work concerns DCPM. In this paper, we have used it as a quality function that is method-independent for assessing the directed core–periphery partition in equation (2.2) produced by different methods. It would be interesting to develop methods which optimize the DCPM quality function directly.

Finally, in future work, it would be interesting to explore more datasets with complex structure. In studies of meso-scale structure (e.g. core–periphery and community structures), there are many possible methods for detecting a given partition structure. While our methods are designed to detect a specific core–periphery structure, empirical networks often contain more than one type of meso-scale structure at a time. Adapting our partition selection process to other types of meso-scale structures and combining different methods to explore a range of meso-scale structures may yield novel insights about empirical networks.

## Supplementary Material

Supplementary Information

## Footnotes

1 For completeness, we display the sample standard deviation for all methods in electronic supplementary material, D.

## Data accessibility

The code has been shared at https://github.com/alan-turing-institute/directedCorePeripheryPaper.
