# Supplementary material for: Core–periphery structure in directed networks
Source: Proc Math Phys Eng Sci. 2020 Sep 9;476(2241):20190783. doi: 10.1098/rspa.2019.0783 (PMC7544362; doi:10.1098/rspa.2019.0783)
Supplement: Supplementary Information [file rspa20190783supp1.pdf]

Core–Periphery Structure in Directed Networks
Supplementary Information

Andrew Elliott[1,2], Angus Chiu[2], Marya Bazzi[1,3,4], Gesine Reinert[1,2] and Mihai Cucuringu[1,2,4]
[1] The Alan Turing Institute, London, UK [2] Department of Statistics, University of Oxford, UK
[3] Mathematics Institute, University of Warwick, UK [4] Mathematical Institute, University of Oxford, UK

A Alternative Definitions of Directed Core-Periphery

In the main text, we proposed a novel directed core–periphery structure, introduced methods to detect it, compared the performance of these methods on synthetic benchmarks, and explored this structure in real-world networks. While we find the extension of core–periphery to the directed case a natural one to consider and insightful in real-world data, there are other possible choices. To better understand the space of possible directed configurations that still maintain a core–periphery-like structure, we propose a set of criteria, and enumerate all possible structures which satisfy them.

We start with a partition of the vertex set into four potentially empty sets, which we denote as Core 1, Core 2, Periphery 1, and Periphery 2 (we suppress the corresponding number when there is only one set of its kind). For a directed core–periphery structure, we impose the following restrictions

1. There cannot be connections within or between periphery sets.
2. A periphery set must connect to exactly one core set.
3. Any pair of sets designated as core must connect to each other, must be connected internally, and each core set must be connected to at least one periphery set.
4. The structure can have no reciprocal edges between any two distinct blocks.

We detail below the structures that satisfy the above assumptions.

(i) 1 Core set and 1 periphery set We first consider the case of one core and one periphery set. As the core must connect internally, and the periphery set must connect to the core, there are two possible design matrices

$$\begin{array}{r|cc|} & \text{Core} & \text{Periphery} \\ \text{Core} & 1 & 1 \\ \text{Periphery} & 0 & 0 \end{array} \tag{A.1}$$

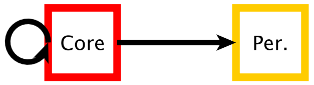

$$\begin{array}{r|cc|} & \text{Core} & \text{Periphery} \\ \text{Core} & 1 & 0 \\ \text{Periphery} & 1 & 0 \end{array} \tag{A.2}$$

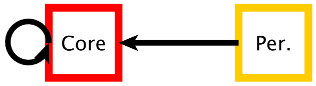

Directed core–periphery structure (A.1) and (A.2) represent an asymmetric flow between core and periphery, as a periphery either only sends or only receives links. This can be a flow of information, such as follower-only accounts in Twitter networks, or a flow of money such as peripheral banks in inter-bank networks who sell their loans to central banks.

(ii) 2 Core sets and 1 periphery set As we restrict the periphery set to connect to only one core, and each core set to connect to at least one periphery, there are no possible design matrices that satisfy these constraints.

(iii) 1 Core set and 2 periphery sets In order for this case to make the structure identifiable, we exclude structures that can be obtained by splitting the peripheries of our 1 core and 1 periphery structures. Under this restriction, only one configuration is possible, namely having the peripheries connect in different directions. Below is the resultant design matrix for this structure.

$$\begin{array}{r|ccc|} & \text{Core} & \text{Per. 1} & \text{Per. 2} \\ \text{Core} & 1 & 0 & 1 \\ \text{Per. 1} & 1 & 0 & 0 \\ \text{Per. 2} & 0 & 0 & 0 \end{array} \tag{A.3}$$

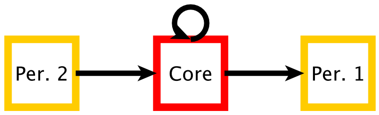

Directed core–periphery structure (A.3) is a particular instance of the well-known bow-tie structure, essentially having a set of vertices that only send material (content, money, etc.), and a set of vertices that only receive material; there are several known real world examples, namely of the internet in [1] and in biological networks [2].

(iv) 2 Core sets and 2 periphery sets In the case of 2 core and 2 periphery sets, the cores must interconnect, and the link cannot be reciprocated, so that there is only one possible structure between the two core sets. By assumption, the periphery vertices only connect to one core set, and each core set must be connected to a periphery. Therefore, there are four possible such structures

	Per. 1	Core 1	Core 2	Per. 2
Per. 1	0	1	0	0
Core 1	0	1	0	0
Core 2	0	1	1	1
Per. 2	0	0	0	0

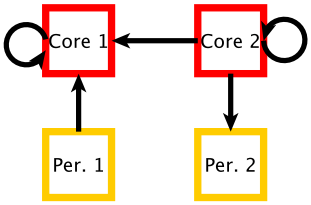

(A.4)

	Per. 1	Core 1	Core 2	Per. 2
Per. 1	0	0	0	0
Core 1	1	1	0	0
Core 2	0	1	1	0
Per. 2	0	0	1	0

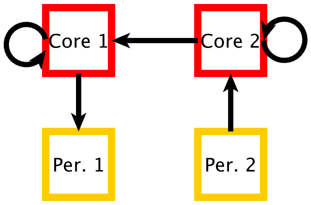

(A.5)

	Per. 1	Core 1	Core 2	Per. 2
Per. 1	0	1	0	0
Core 1	0	1	0	0
Core 2	0	1	1	0
Per. 2	0	0	1	0

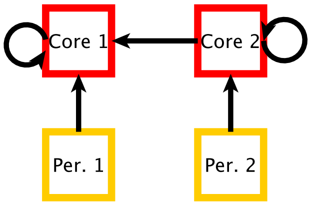

(A.6)

	Per. 1	Core 1	Core 2	Per. 2
Per. 1	0	0	0	0
Core 1	1	1	0	0
Core 2	0	1	1	1
Per. 2	0	0	0	0

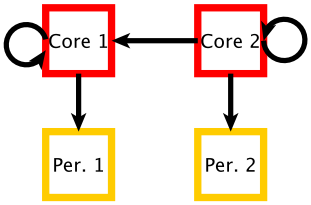

(A.7)

To the best of our knowledge, these structures have not been directly studied before. The directed core–periphery structure (A.5) is essentially a bow-tie in which the core can be split into two sets, one which takes the incoming connections of the periphery vertices, and the remaining core set which sends links to the additional periphery set. This could be seen as a two-step process version of the bow-tie structure, in which material is taken in, processed, and then distributed to a second set that performs a similar action. In contrast, the structure (A.4), which is the one on which the main paper focuses, is not a simple extension of the bow-tie structure due to the fact that, contrary to bow-tie, the flow in (A.4) is not uni-directional.

B Selection and Performance of Different Variants of the HITS Methods

Several methodological choices were made during the development stage of the HITS and AdvHits methods, based on their relative performance on our synthetic benchmarks. The Hits method can be divided into the following steps.

1. Construct the Hits scores of the observed graph adjacency matrix.
2. Construct vertex scores from the low-rank approximation.
3. Cluster the vertices by applying k-means++ to the scores.

For the scoring step described in the main text, we use the scores $\mathbf{C}_{In}^{HITS}$, $\mathbf{C}_{out}^{HITS}$, $\mathbf{P}_{In}^{HITS}$ and $\mathbf{P}_{Out}^{HITS}$. This formulation is purely score-based, and it does not leverage other network information such as the score of the neighbours of a vertex. In this section, we detail an additional scoring variant we considered. For the first alternative formulation, heuristically, a vertex i should have a high $\mathcal{P}_{in}$ score if

1. it has in-coming edges from vertices that have high $\mathcal{C}_{out}$ scores and does not have in-coming edges from vertices that have high $\mathcal{C}_{in}$ scores;
2. it does not have many out-going edges to nodes with high $\mathcal{C}_{out}$ or $\mathcal{C}_{in}$ scores.

Additionally, a vertex i should have a high $\mathcal{P}_{out}$ score if

1. it has out-going edges to vertices that have high $\mathcal{C}_{in}$ scores, and does not have out-coming edges to vertices that have high $\mathcal{C}_{in}$ scores;
2. it does not have many in-coming edges from nodes with high $\mathcal{C}_{out}$ or $\mathcal{C}_{in}$ scores.

Combining these, we propose the following alternative scoring scheme

$$P_{In}^{HITS-Alt}(u) = \sum_{v=1, v\neq u}^{n} \left(-A_{uv}C_{In}^{HITS}(v) - A_{uv}C_{Out}^{HITS}(v) - A_{vu}C_{In}^{HITS}(v) + A_{vu}C_{Out}^{HITS}(v)\right), \tag{B.8}$$

$$P_{Out}^{HITS-Alt}(u) = \sum_{v=1, v\neq u}^{n} \left(A_{uv}C_{In}^{HITS}(v) - A_{uv}C_{Out}^{HITS}(v) - A_{vu}C_{In}^{HITS}(v) - A_{vu}C_{Out}^{HITS}(v)\right). \tag{B.9}$$

To decide which approach to use in the main paper, we considered both scoring schemes, and selected the scoring scheme in the main body as it outperforms the alternatives on all three benchmarks. We also considered two variants of the HITS method, based on the alternative scoring in Eqs. (B.8) and (B.9). The scoring scheme we presented in the main body outperforms the alternatives on all three benchmarks.

C Stochastic Block Model Fitting: The MaxLike Method

In this section, we detail the algorithms for fitting a stochastic block model to the proposed structure. We implemented MaxLike from [3] as follows.

1. Assign all vertices to a random set (CurrentPartition).
2. Calculate the likelihood of the random partition, and store it and the partition itself in BestPartition.
3. Make a list List1 of all vertices.
4. Find the vertex in List1 for which the movement to a new set will increase the likelihood the most (or decrease the least). Perform the move and remove the vertex from List1.
5. If the likelihood of the current partition is larger than BestPartition, then replace BestPartition with the current partition.
6. If List1 is not empty go to Step **4**.
7. If BestPartition has improved since Step **3**, then set CurrentPartition to BestPartition and go to Step **3.**
8. Return BestPartition.

D Synthetic Benchmarks

In this section, we present additional content and results which relate to our synthetic benchmarks.

(a) Similarity Measures

Adjusted Rand index The Adjusted Rand Index (ARI) [4] measures the similarity between two partitions of a data set. Let $V = (v_1, v_2, \dots, v_n)$ be a set of vertices we wish to cluster, let $X = (X_1, X_2, X_3, X_4)$ be a partition of V into four clusters, and let $Y = (Y_1, Y_2, Y_3, Y_4)$ be the ideal partition. We let a be the number of vertex pairs that are placed in the same set of X and in the same set of Y; b be the number of vertex pairs that are placed in the same set of X but in different sets of Y; c be the number of vertex pairs that are placed in different sets of X but in the same set of Y; d be the number of vertex pairs that are placed in different sets of X and in different sets of Y. The Adjusted Rand Index [4] is

$$\text{ARI} = \frac{\binom{n}{2}(a+d) - [(a+b)(a+c) + (c+d)(b+d)]}{\binom{n}{2}^2 - [(a+b)(a+c) + (c+d)(b+d)]}.$$

The Adjusted Rand Index (ARI) is bounded by ± 1 with an expected value of 0. An ARI close to 1 indicates an almost perfect match between the two partitions, whereas an ARI close to -1 indicates that the agreements between two partitions is less than what is expected from random labellings (which has an ARI of 0) and points towards complementary partitions.

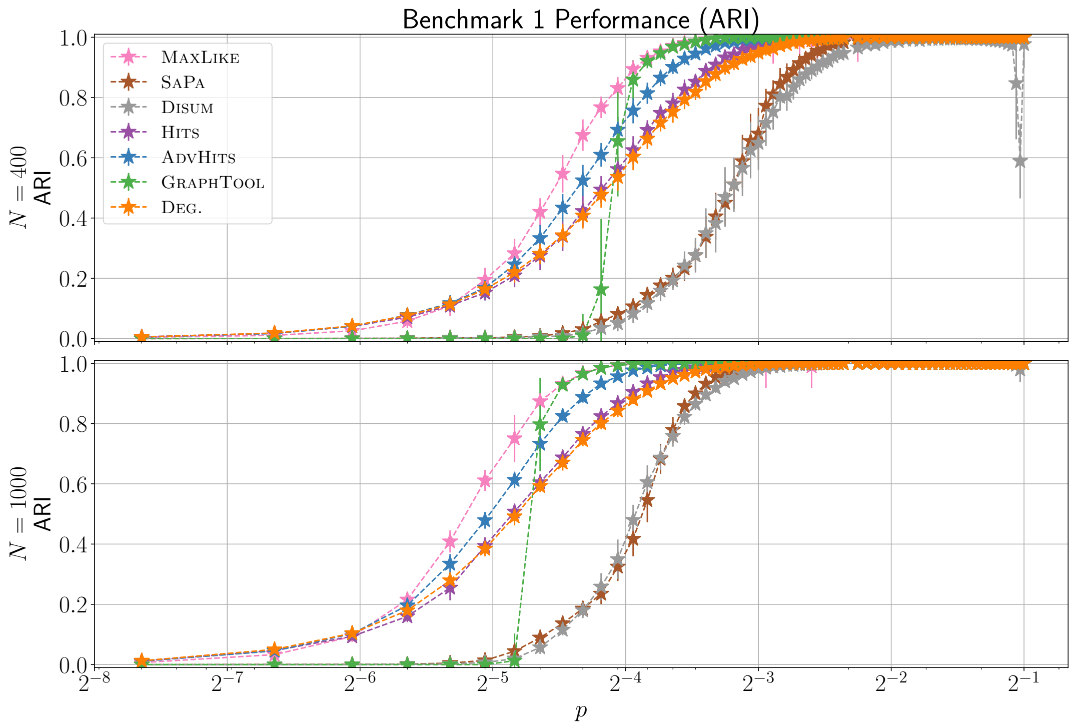

Figure SI 1: Performance on Benchmark 1. The ARI between the planted partition of the graph and the partition detected by each method, with the upper, respectively lower, panel showing results for networks of size $n = 400$, and $n = 1000$ (Benchmark 1, Benchmark 2). On the x axis, we vary the parameter p on a log scale. Error bars are one sample standard deviation. For a large range of values, Deg. and Hits are barely distinguishable.

Normalised Mutual Information (NMI) and Variation of Information (VOI) These measures are based on the concept of entropy and mutual information [5]. For a random variable X with values in a discrete set $\mathcal{S}_X$, and a random variable Y with values in a discrete set S_Y [5], the entropy of X is $H(X) = -\sum_{a \in S_X} P(X = a) \log(P(X = a))$, and the mutual information $MI(X, Y)$ of X and Y is given by

$$MI(X,Y) = \sum_{a \in S_X} \sum_{b \in S_Y} P(X = a, Y = b) \log \left(\frac{P(X = a, Y = b)}{P(X = a)P(Y = b)} \right).$$

The variation of information $VOI(X, Y)$ is defined as

$$VOI(X,Y) = H(X) + H(Y) - 2MI(X,Y),$$

see for example [6]. Thus, if $MI(X,Y) = H(X) = H(Y)$, indicating that the information in X is perfectly captured by the information in Y, $VOI(X,Y)$ would have a value of 0. If $MI(X,Y) = 0$, then $VOI(X,Y)$ takes the largest possible value. Normalised mutual information $NMI(X,Y)$ is defined in [7] as

$$NMI(X,Y) = \frac{2MI(X,Y)}{H(X) + H(Y)}.$$

Again, if $H(X) = H(Y) = MI(X,Y)$, then $NMI(X,Y) = 1$, whereas if $MI(X,Y) = 0$, then $NMI(X,Y) = 0$.

Next we show the in-depth results on Benchmark 1, Benchmark 2 and Benchmark 3, supporting the claims and results in the main document.

(b) Additional Results for Benchmark 1

Fig. SI 1 shows the ARI between the planted partition of the graph and the partition detected by each method, using the Benchmark 1 data set. Error bars denote one sample standard deviation. MaxLike has the highest ARI across a wide range of intermediate values for the parameter p, followed by AdvHits. The peformance of Deg. and Hits are very similar across a wide range of values.

The NMI and VOI results for Benchmark 1 can be found in Fig. SI 2 and in Table 1 and Table 2. Broadly, the results are qualitatively similar to the ARI results. For both NMI and VOI, SaPa2 and DiSum are outperformed by all other

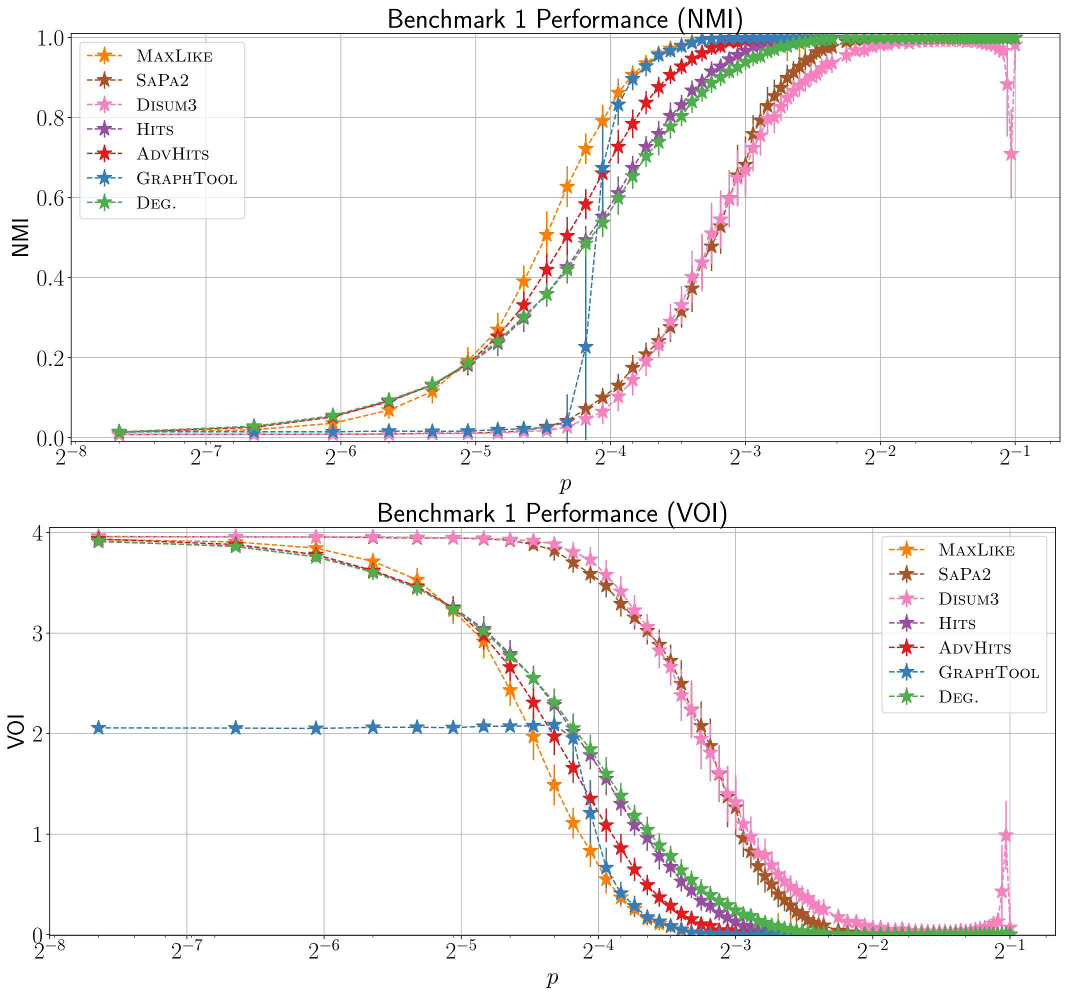

Figure SI 2: Performance on Benchmark 1. The NMI (top panel) and VOI (bottom panel) between the underlying partition of the graph and the detected partition for $n = 400$. On the x axis, we vary the parameter p, with values close to 0 more difficult to detect than higher values of p. Error bars are one sample standard deviation.

methods, which is consistent with the results of ARI. Similarly AdvHits and MaxLike outperform clustering based on degree in all cases, with HITS performing at least as well as clustering based on degree. We also observe an equivalent performance result for AdvHits, with an improvement over the HITS method, but outperformed by MaxLike.

NMI table p	0.4	0.1	0.09	0.08	0.07	0.06	0.05	0.04	0.03	0.02
Deg	**1.0**	0.864	0.804	0.739	0.654	0.537	0.42	0.303	0.186	**0.0937**
SaPa	**1.0**	0.438	0.316	0.242	0.175	0.1	0.0421	0.0172	0.0112	0.00913
Disum	0.992	0.438	0.332	0.232	0.145	0.0649	0.0271	0.0153	0.0105	0.00883
GraphTool	**1.0**	0.994	0.979	0.957	0.897	0.674	0.04	0.0224	0.0164	0.0164
Hit	**1.0**	0.89	0.831	0.759	0.674	0.552	0.426	0.299	0.181	0.0896
AdvHits	**1.0**	0.965	0.931	0.877	0.788	0.658	0.5	0.325	0.181	0.0902
MaxLike	**1.0**	**0.995**	**0.98**	**0.96**	**0.907**	**0.791**	**0.627**	**0.391**	**0.192**	0.069

Table 1: Average NMI of the methods under comparison on Benchmark 1 ($DCP(1/2 + p, 1/2 - p)$) for different values of p, and with network size $n = 400$. The largest values for each column are given in boldface.

We highlight the VOI results for GraphTool. It outperforms the other methods for $p < 2^{-4}$, whereas ARI and NMI show the opposite performance. This discrepancy may be related to the effect which placing almost all vertices into one set has on the similarity measures. Indeed, Table 3 shows the average size of the largest set in AdvHits and GraphTool. For AdvHits, the average largest set size is close to an equal division into four equally-sized sets, whereas GraphTool incorrectly places most of the vertices in a single set for $p < 0.06 \approx 2^{-4.06}$. With most vertices in the GraphTool partition in one set, the entropy will be close to 0, as $-\log(1-\epsilon) \approx 0$. As mutual information is by definition less than the

VOI Table p	0.4	0.1	0.09	0.08	0.07	0.06	0.05	0.04	0.03	0.02
DEG	**0.0**	0.546	0.783	1.04	1.38	1.85	2.31	2.77	3.24	3.61
SAPA	**0.0**	2.24	2.72	3.02	3.29	3.59	3.82	3.92	3.95	3.95
DISUM	0.0311	2.24	2.66	3.06	3.41	3.73	3.88	3.93	3.95	3.96
GRAPHTOOL	**0.0**	0.022	0.0857	0.174	0.412	1.21	2.09	**2.07**	**2.06**	**2.06**
HITS	**0.0**	0.442	0.675	0.962	1.3	1.79	2.29	2.79	3.26	3.62
ADVHITS	**0.0**	0.154	0.288	0.494	0.861	1.35	1.97	2.66	3.25	3.62
MAXLIKE	**0.0**	**0.0196**	**0.0797**	**0.159**	**0.371**	**0.834**	1.49	2.43	3.23	3.71

Table 2: Average VOI of the methods under comparison on Benchmark 1 ($DCP(1/2+p, 1/2-p)$) for different values of p, and with network size $n = 400$. The largest values for each column are given in boldface. Numbers are to 3sf.

n	p	ADVHITS	GRAPHTOOL
400	0.4	102.1	100.42
400	0.09	103.28	101.24
400	0.08	105.52	102.12
400	0.07	109.06	103.98
400	0.06	114.38	150.5
400	0.05	117.5	387.22
400	0.04	120.1	394.56
400	0.03	116.12	396.0
400	0.02	114.22	395.88

Table 3: Average size of the largest set in the detected partition in Benchmark 1. Note that the maximum achievable set size for a division into four non empty sets is $400 - 1 - 1 - 1 = 397$.

entropy, the VOI thus becomes close to the entropy of the almost constant sequence, which is equal to $-\log_2(0.25) = 2$, a relatively high value. The behaviour of NMI on GRAPHTOOL can also be similarly explained; as mutual information is close to 0, NMI also becomes close to 0.

(c) Additional Results for Benchmark 2

Plots for Benchmark 2, which show the combination of parameters p_1 and p_2 that yield an ARI of 0.75, or 0.9, respectively, are shown in Fig. SI 3. The results are broadly consistent, with similar performance for many regimes in the 0.9 contour, but a larger performance difference in the 0.75 contour. The 0.9 contour (Fig. SI 3) shows that the performance of GRAPHTOOL is closer to that of the likelihood methods compared to the 0.75 contour. This could be related to GRAPHTOOL often placing all nodes in the same set when the structure is weak.

Fig. SI 4 shows contour plots for NMI and VOI for network size $n = 400$; the results for $n = 1000$ are broadly similar. The NMI contours give the same ordering and roughly the same performance as observed for ARI. The VOI contours, displayed in Fig. SI 4 right panel, show broadly consistent findings but with a few small deviations.

(d) Additional Results for Benchmark 3

The average ARI results as well as the NMI and VOI results of all methods on Benchmark 3 can be found in Fig. SI 5. We fix the size of three sets at $n_1 = n/4 = 100$ and vary the size of the final set n_2. For example, to vary the size of $\mathcal{P}_{out}$, we fix $n_{\mathcal{C}_{in}} = n_{\mathcal{C}_{out}} = n_{\mathcal{P}_{in}} = n_1$ and test performance when we let $n_{\mathcal{P}_{out}} = n_2 \in \{2^{-3}n_1, 2^{-2}n_1, \dots, 2^3 n_1\}$, with equivalent formulations for the other sets. Thus for $\frac{n_2}{n_1} = 1$ we have equal-sized sets, which is equivalent to the model in Benchmark 1, for $\frac{n_2}{n_1} > 1$ one set is larger than the remaining sets, and for $\frac{n_2}{n_1} < 1$, one set is smaller than the others.

The results are broadly consistent across each of the similarity measures, although there is a small deviation with the performance of ADVHITS for $\frac{n_2}{n} > 2$; the information theoretic measures (NMI and VOI) yield a slightly higher performance for ADVHITS than ARI. In both cases, the performance of ADVHITS collapses in this region.

The performance of the methods differs depending on whether it is a core set or a periphery set which changes size. In the periphery, smaller set sizes seem to have a minimal impact on performance, while large set sizes lead to a large decay in performance. In the core, both smaller and larger sets appear to have a significant impact.

Summarising, in Benchmarks 1 and 2, the results are qualitatively similar between the graph sizes, with methods failing in the same order and in the same manner, although the range of values where this failure occurs shifts. This is expected as there is additional information present in larger graphs, so we would expect smaller values of p to have potentially

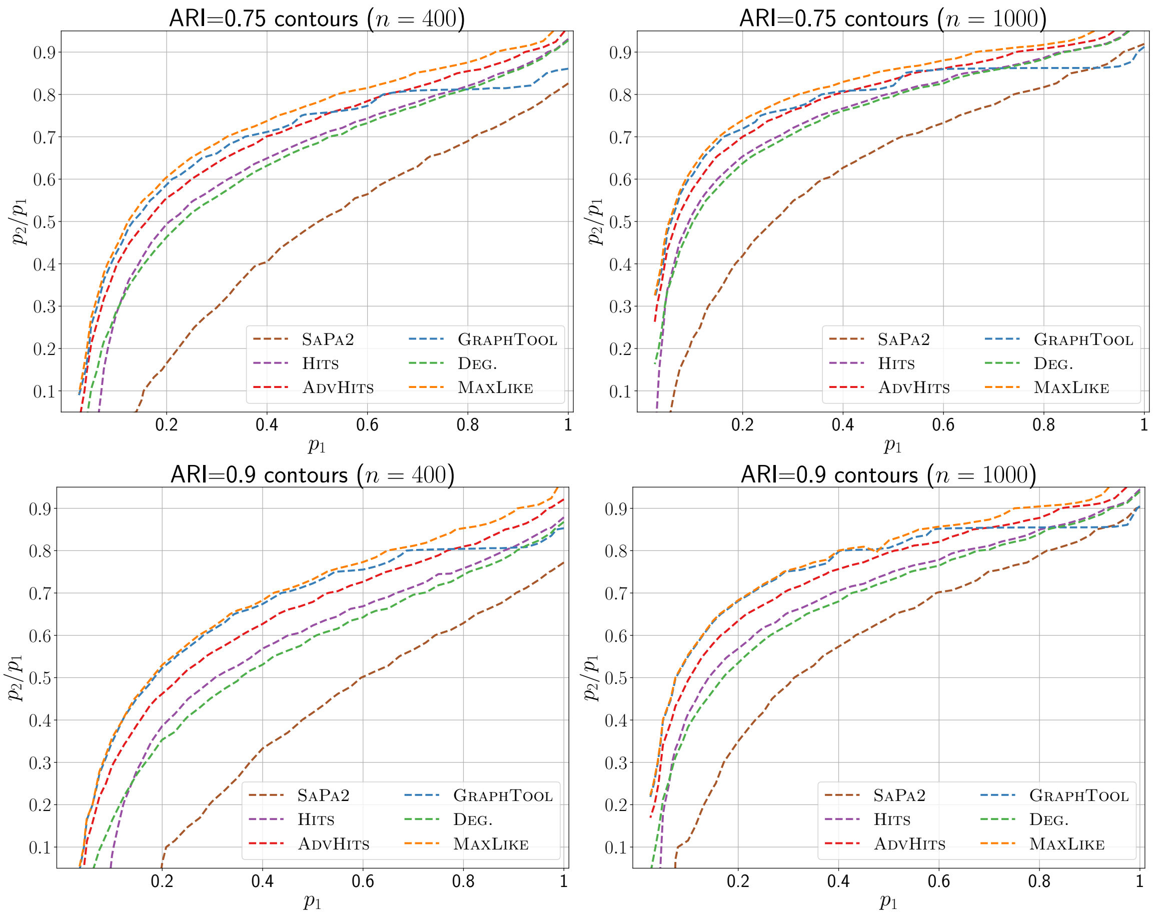

Figure SI 3: Contour plot of ARI=0.75 and ARI=0.9 on Benchmark 2. For this plot, we calculate the average ARI of size 400 (left panel 50 repeats) and 1000 (right panel 10 repeats) varying $p_1 \in \{0.025, 0.05, \ldots, 1.0\}$ and $\frac{p_2}{p_1} \in \{0, 0.05, 0.1, \ldots, 0.95\}$, and then display the contours corresponding to an average ARI of 0.75 or 0.9, respectively. We note that the fast method HITS outperforms the comparisons Deg. and SaPa2, although they are hard to distinguish due to similar performance. AdvHits also outperforms the fast comparisons. For moderate values of p
, MaxLike has similar performance to GraphTool, but like AdvHits outperforms GraphTool for larger values of p_1.

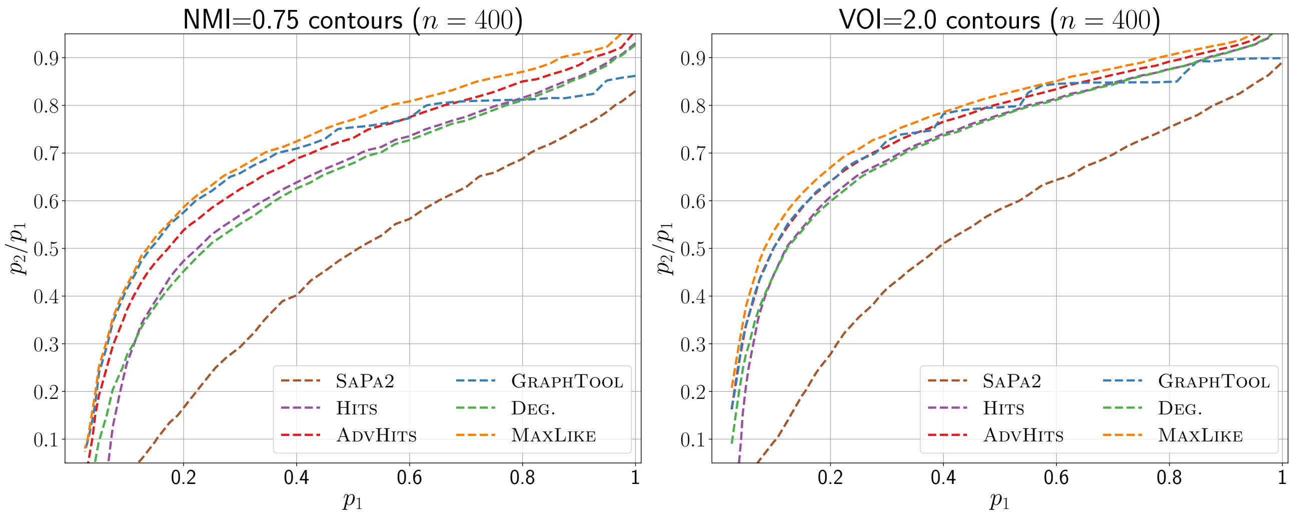

Figure SI 4: Contour plot of NMI=0.75 (left) VOI=2.00 (right) on the Benchmark 2. For this plot, we calculate the average VOI over 50 networks of size 400 varying $p_1 \in \{0.025, 0.05, \ldots, 1.0\}$ and $\frac{p_2}{p_1} \in \{0, 0.05, 0.1, \ldots, 0.95\}$, and then display the contours corresponding to an average NMI= 0.75 (left panel) VOI= 2.0 (right panel).

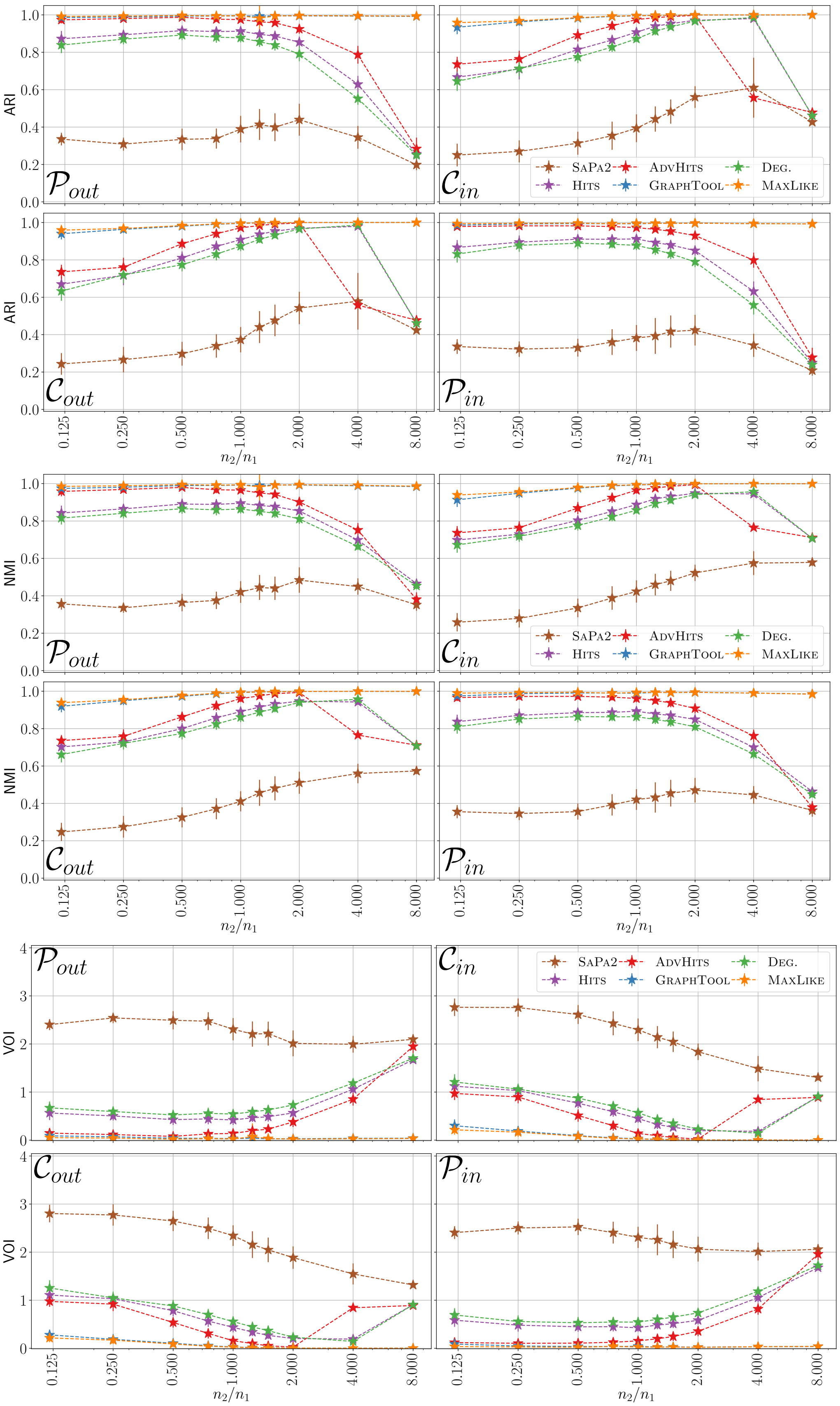

Figure SI 5: Performance of all methods on Benchmark 3, with sets of different sizes using ARI (top panel), NMI (middle panel) and VOI (bottom panel). We fix $p = 0.1$ and the size of 3 sets at $n_1 = n/4 = 100$, and vary the size of the final set (n_2) and measure the performance. The set varied in each panel is displayed in bold in each panel. We use 50 repeats for each point, and the error bars are 1 sample standard deviation.

larger effects on performance (up to detectability limits). We observe a similar effect in Benchmark 3 for $n = 1000$; with the increased network size (and fixed value of $p = 0.1$), with many methods performing better over the range of block sizes, although we observe a similar decay in the performance of AdvHits.

(e) Performance of DiSum and SaPa variants

As alluded to in the main text, we consider a small number of variants of our spectral comparison methods and only display the best performing method. In this section we discuss the other variants we considered on each of our benchmarks.

SaPa **Variants** From [8], SaPa employs a similarity measure using two different types of symmetrisations for directed (asymmetric) adjacency matrices, namely bibliometric symmetrisation and degree discounted symmetrisation. We use them here to construct two Sapa variants, namely SaPa1 and SaPa2, which we then cluster. They are defined as

1. SaPa1 - Bibiometric symmetrisation: $A_I A_I^T + A_I^T A_I$,
2. SaPa2 - Degree discounted: $D_o^{-0.5} A D_i^{-0.5} A^T D_o^{-0.5} + D_i^{-0.5} A D_o^{-0.5} A^T D_i^{-0.5}$,

where D_i is a diagonal matrix with the in-degrees on the diagonal, D_o is an diagonal matrix with the out-degrees on the diagonal, $A_I = A + I$, where I is the identity matrix. In the original formulation of SaPa, these similarity matrices were then clustered via various clustering algorithms. Instead, we use the same kmeans++ pipeline used previously.

DiSum **Variants** The second fastest approach, DiSum from [9], clusters the graph using the left and right singular vectors of the regularised and degree normalised adjacency matrix $((k_u^{out} + \frac{m}{n})(k_v^{in} + \frac{m}{n}))^{-1/2} A_{uv}$. While [9] performed a column and row clustering, as we require clustering of the whole network, we consider four different variants of this approach, as follows.

1. DiSum1: A row clustering into 4 sets,
2. DiSum2: A column clustering into 4 sets,
3. DiSum3: A combined row and column clustering into 4 sets (by concatenating the left and right singular vectors),
4. DiSum4: Combining a row clustering into 2 sets with a column clustering into 2 sets to obtain 4 resultant sets (with each set defined by the intersection of a pair of sets from the different clusterings). Note, we use a separate row and column clustering rather than the combined clustering in [9].

We again cluster the resulting vectors using our standard k-means++ pipeline.

Results We observe that SaPa2 and DiSum3 outperform their counterparts on Benchmark 1 and Benchmark 2. In Benchmark 3, DiSum4 outperforms DiSum3, for large differences in group sizes. However, given that DiSum3 performs reasonably and outperforms DiSum4 considerably in Benchmarks 1 and 2, we select DiSum3 as our overall DiSum method.

(f) Testing our Novel Quality Measures

In this section, we present additional results testing the relationship between our quality measures and the ground truth partition. We display the average *DCPM* of the partitions from Benchmark 1 in the main body (Table 4). While the method with the highest average *DCPM* does not always have the highest ARI, its ARI is often close to the highest value, i.e. $p = 0.09$, GraphTool has the highest average *DCPM*, with an ARI of 0.968, whereas the highest ARI is 0.987, only a marginal difference. For completeness, in Table 5, we also present the sample standard deviation of the p-value, *DCPM* and ARI. The sample standard deviation is often many orders of magnitude below the average for the p-values, and typically, at least one order of magnitude below for DCPM and ARI.

(g) Run Time On Synthetic Networks

The two panels of Fig. SI 6 show the run time of each of the methods on Benchmark 1. We note that the time comparison is advisory, as this calculation was performed on an Azure Virtual Machine. To mitigate the effects of hyperthreads, we used half of the available vCPUs, but we cannot control for other users present on the same physical hardware. Further, different approaches are implemented in different languages, with the matrix-based methods (HITS, AdvHits, SaPa, DiSum, and HITS) being implemented in Python using NumPy and SciPy vectorised matrix operations, and GraphTool in C++ with Python wrapping[1]. Finally, the likelihood methods are implemented in pure Python, ran under cPython, with the exception of MaxLike for $n = 1000$ which was ran under PyPy [10]. When testing these methods under PyPy, we obtain a $\approx$ 10x speed up on the cPython times. The implementations of SaPa, DiSum, and HITS are relatively naïve, and could be improved, for example, by exploiting the specialised matrix construction code used in AdvHits.

We observe that the performance of many of the methods is relatively constant with respect to p, with an increase in run time when p is small. This behaviour is especially pronounced in AdvHits with comparatively fast times for the

[1]The GraphTool timings also contains the C++ graph construction, and the matrix approaches timings contain the matrix construction

p	0.4	0.1	0.09	0.08	0.07	0.06	0.05	0.04	0.03	0.02
Deg	**0.491**	**0.0996**	0.0778	0.0676	0.057	0.0466	0.0381	0.0295	0.0151	0.00877
SaPa	**0.491**	0.0535	0.0331	0.0226	0.0155	0.00876	0.00384	-0.00184	0.000549	-0.00104
Disum	0.49	0.0487	0.0331	0.0202	0.00952	0.00693	0.00169	0.0013	0.00295	-0.00194
GraphTool	**0.491**	0.0933	**0.0829**	**0.0736**	**0.064**	0.05	0.00217	0.000524	0.000127	-3.66e-06
Hits	**0.491**	0.0911	0.0796	0.069	0.0587	0.0485	0.0385	0.0304	0.0157	0.0141
AdvHits	**0.491**	0.0932	0.0828	0.0734	0.0635	0.0541	0.0451	0.0347	0.0216	0.00808
MaxLike	**0.491**	0.0932	0.0828	**0.0736**	0.0639	**0.055**	**0.0472**	**0.0404**	**0.0355**	0.0128

Table 4: Average $DCPM$ of the methods under comparison on Benchmark 1 ($DCP(1/2+p, 1/2-p)$) for different values of p and with network size $n = 400$. The largest values for each column are given in boldface. For each measure, we reassign the set labels based on the log likelihood of a DCP with fitted p_1 and p_2. We perform this correction to account for the fact that the comparison methods do not explicitly give allocations to our named sets.

p	0.1				0.04				0.02			
	p-value				p-value				p-value			
	ER	Con.	$DCPM$	ARI	ER	Con.	$DCPM$	ARI	ER	Con.	$DCPM$	ARI
Hits	8.9e-19	8.9e-19	0.00206	0.0217	0.00178	0.000891	0.00321	0.0469	0.354	0.434	0.0187	0.0144
AdvHits	8.9e-19	8.9e-19	0.0016	0.0102	0.0151	0.0196	0.00416	0.0551	0.315	0.416	0.0186	0.0221
MaxLike	8.9e-19	8.9e-19	0.00158	0.00453	8.9e-19	8.9e-19	0.00128	0.056	0.459	0.496	0.0319	0.0265

Table 5: Sample standard deviation of the p-value (ER and configuration model, Con.), $DCPM$ and ARI, by method and parameter in a $DCP(1/2+p, 1/2-p)$ model; p-values are rounded to 3 sf.

easier graphs, and slower times for the harder graphs, which is likely to be due to the fact that the method falls back to the single vertex update scheme for small values of p. This is also likely to be related to the error bars (one sample standard deviation), which increase for low p in AdvHits for $n = 400$. We note that AdvHits is slower than Hits when p is small and has a better time complexity than the PyPy and non-PyPy versions of MaxLike (with 10 repeats), but may be closer in performance to a simpler likelihood optimiser highlighting that the AdvHits approach is a medium speed approach in contrast to the fast approaches.

E Political Blogs Results

The *PolBlogs* data set from [11] consists of political blogs as nodes, and a directed edge from one blog to another denoting that the first blog contains at least one link to the second blog. The data set was collected on a single day during the 2004 US presidential election. After collapsing 65 multi-edges, it contains 1,490 nodes and 19,090 edges. The set of blogs is divided into 758 liberal blogs and 732 conservative blogs. For the analysis in this section, we focus on the largest weakly connected component of the network, which has $n = 1,222$ nodes and 19,024 edges.

The right panel of Fig. SI 7 shows the ARI between the partitions given by 11 runs (original partition with 10 additional repeats) for each of our methods and a single run of BowTie. We note a high within-method AR and a high similarity between AdvHits and MaxLike. Furthermore, and importantly, there is a low ARI between our methods and BowTie, indicating that our methods do uncover a different structure.

Inspecting the left panel of Fig. SI 7, the HITS method gives p-values lower than 5% against the directed ER null model, but not against the directed configuration model, potentially indicating that the recovered structures can be modelled as a function of the in- and out-degree distributions. The other methods yield partitions with p-values less than 5% on both tests. Considering the DCPM, we note that the MaxLike partition has the highest value, and thus we focus on this structure for the remainder of the analysis.

The structure uncovered with MaxLike, shown in Fig. SI 8A, presents both a network visualisation and a matrix showing the density of edges between each pair of sets. We note that there is a visible 'L'-shape structure, indicating a split into the four roles. Fig. SI 8B shows the degree patterns of each set, which broadly matches our intuition from the 'L'-shaped structure. It is notable that not all nodes with very high out-degree are in C_{out}, although all four nodes in C_{out} are in the top 4% of nodes by out-degree.

To validate the structure, we first explore the relationship between the sets we uncover and the political division of the network. Figure SI 8C gives the confusion matrix between each of our divisions and the political division. We observe the sets divide into two roughly similarly-sized sets. These sets are not in 1-1 relationship with the Left/Right party division of the blogs. Hence there is evidence that similar to the division in [12], our partition relates to the role the sets play in the network.

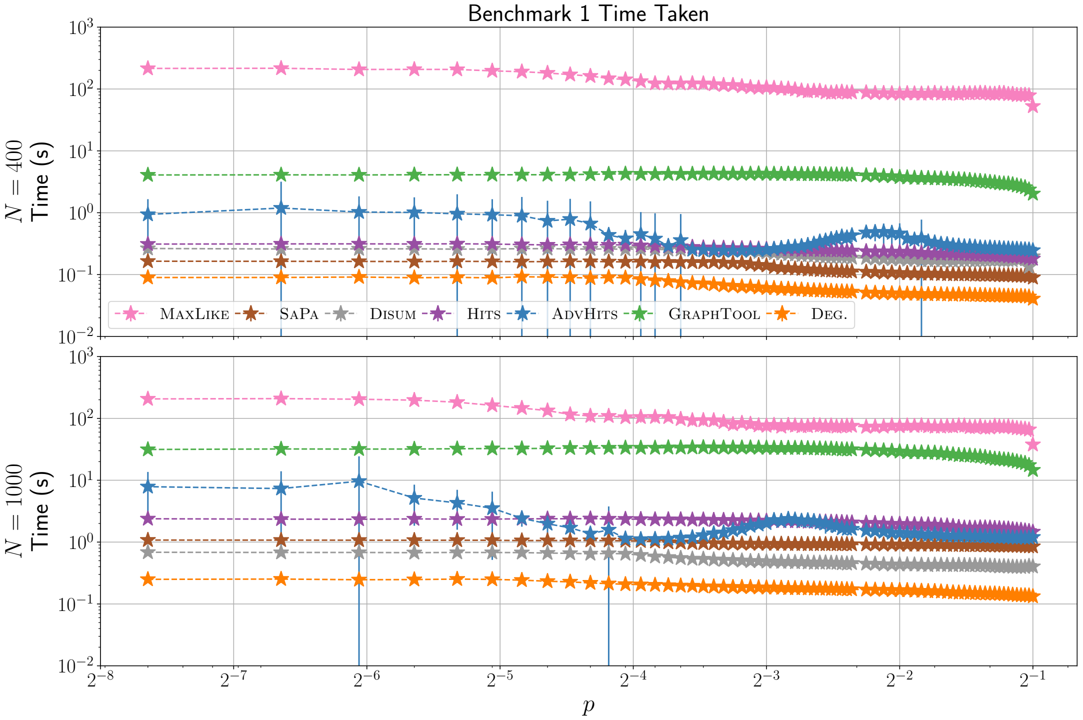

Figure SI 6: Running time of all methods on Benchmark 1 with the upper, respectively lower, panel showing results for networks of size $n = 400$, respectively $n = 1000$. On the x axis, we vary the parameter p, with values close to 0 more difficult to detect than higher values of p. The timing for MaxLike for $n = 1000$ was ran under PyPy, a faster Python interpreter, to highlight the possible speed up.

	Political Blog network		
	p-value		
	ER	Config	DCPM
HITS	0.004	0.960	0.384
AdvHits	0.004	0.004	0.594
MaxLike	0.004	0.004	**0.652**

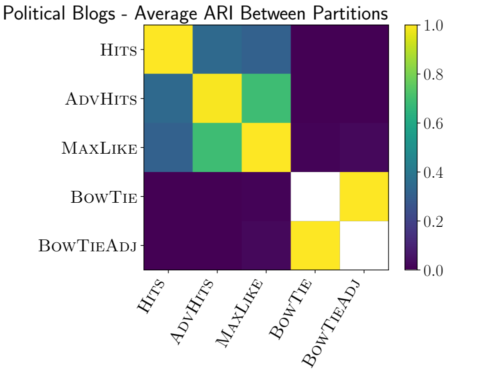

Figure SI 7: *PolBlogs* results. (**Left**) p-values and DCPM values for each partition in the Political Blog data. (**Right**) The ARI between the partitions uncovered by each method. For ease of comparison, values < 0 (i.e. very dissimilar) are set to 0. For the faster methods we compare with 11 runs and show the average similarity between all pairs of partitions; for the slow methods, we use a single run and thus display a blank (white) square. To compare to bow-tie, we compare both to the raw partition into 7 sets and a partition formed by a subset of the nodes corresponding to the main three sets.

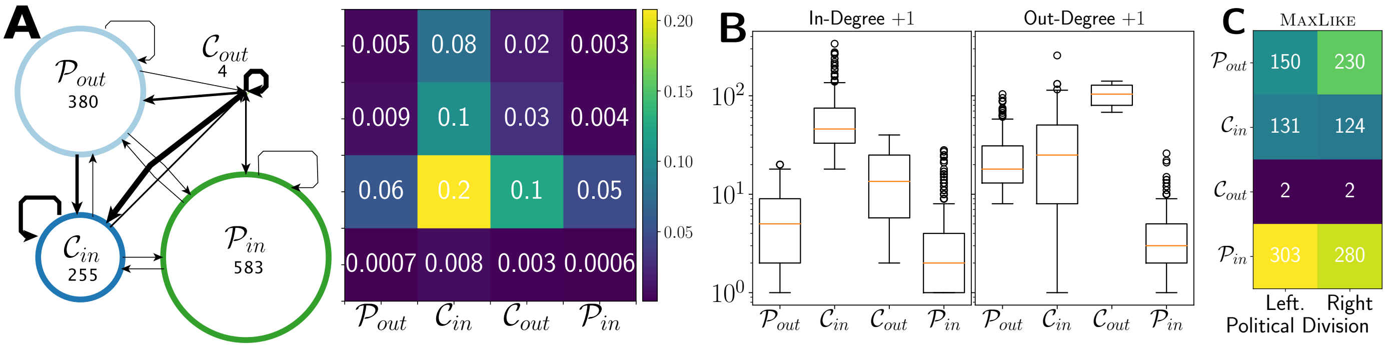

Figure SI 8: Structure in the *PolBlogs* data. We focus on the MaxLike partition as it has the highest DCPM. In **A**, we show summary network diagrams of the structure. In the first summary, the size of each of the nodes is proportional to the number of nodes in the corresponding set, and the width of the lines is given by the percentage of edges that are present. The second summary displays the percentage of edges present between each pair of blocks, allowing for a visualisation that highlights the 'L'-structure. In **B**, we present the in- and out-degree distributions of each of the groups. Finally, in **C**, we compare our division into four groups with the political division of the blogs.

A possible interpretation of the directed core–periphery structure is that the $\mathcal{C}_{in}$ set represents blogs that are seen as authorities, and thus do not link to many other blogs (but are linked to themselves), whereas the opposite is true of those in $\mathcal{C}_{out}$. A potentially related structure was observed in the 15-block SBM from [13], in which blocks are identified that cite but are not cited, and other blocks which are highly cited either in general or by specific groups. An inspection of the web-address corresponding to the nodes in each set suggests that certain sets are enriched for 'blogspot' sites. A 'blogspot' site is a free blogging site that requires less expertise to set up compared to a full website. We see such an authority set in the MaxLike partition; the fraction of 'blogspot' sites is 0.21 in $\mathcal{C}_{in}$ versus > 0.43 in all other sets. Moreover, 39 out of 40 top blogs are in $\mathcal{C}_{in}$ in this division. Moreover, [11] provides a list of the top 20 conservative and the top 20 liberal blogs, by filtering the top ≈ 100 liberal and conservative blogs by degree, and ranking the top blogs by the number of citations of blog posts in an external index (see [11] for details). In MaxLike, all except one of the top blogs are also in $\mathcal{C}_{in}$, thus providing evidence for our hypothesis that $\mathcal{C}_{in}$ is an authority set.

In conclusion, our method finds a division of the *PolBlogs* data in an 'L'-based structure. We further investigated this structure, and found that our $\mathcal{C}_{in}$ set appears to represent authorities, in contrast to the $\mathcal{C}_{out}$ which represents non-authority core nodes. We validate this split using two covariates, first the presence of 'blogspot' website and the second using the list of top blogs provided by [11].

References

[1] Broder A, Kumar R, Maghoul F, Raghavan P, Rajagopalan S, Stata R, Tomkins A, Wiener J. 2000 Graph structure in the Web. *Comput. Netw.* pp. 309–320.

[2] Ma HW, Zeng AP. 2003 The connectivity structure, giant strong component and centrality of metabolic networks. *Bioinformatics* **19**, 1423–1430.

[3] Karrer B, Newman ME. 2011 Stochastic blockmodels and community structure in networks. *Phys. Rev. E* **83**, 016107.

[4] Hubert L, Arabie P. 1985 Comparing partitions. *J Classif* **2**, 193–218.

[5] Cover TM, Thomas JA. 2006 *Elements of Information Theory (Wiley Series in Telecommunications and Signal Processing)*. New York, NY, USA: Wiley-Interscience.

[6] Meilă M. 2007 Comparing clusterings—an information based distance. *J. Multivar. Anal.* **98**, 873 – 895.

[7] Kvalseth TO. 1987 Entropy and correlation: Some comments. *IEEE Transactions on Systems, Man, and Cybernetics* **17**, 517–519.

[8] Satuluri V, Parthasarathy S. 2011 Symmetrizations for clustering directed graphs. In *Proc. of the 14th International Conference on Extending Database Technology* pp. 343–354. ACM.

[9] Rohe K, Qin T, Yu B. 2016 Co-clustering directed graphs to discover asymmetries and directional communities. *Proc. Natl. Acad. Sci. U.S.A.* **113**, 12679–12684.

[10] Bolz CF, Cuni A, Fijalkowski M, Rigo A. 2009 Tracing the Meta-level: PyPy's Tracing JIT Compiler. In *Proc. of the 4th Workshop on the Implementation, Compilation, Optimization of Object-Oriented Languages and Programming Systems* ICOOOLPS '09 pp. 18–25 NY, USA. ACM.

[11] Adamic LA, Glance N. 2005 The political blogosphere and the 2004 US election: divided they blog. In *Proc. of the 3rd international workshop on Link discovery* pp. 36–43. ACM.

[12] Zhang X, Martin T, Newman ME. 2015 Identification of core-periphery structure in networks. *Phys. Rev. E* **91**, 032803.

[13] Peixoto TP. 2014 Hierarchical Block Structures and High-Resolution Model Selection in Large Networks. *Phys. Rev. X* **4**, 011047.
